# Plant-Derived Terpenes as Emerging Therapeutics Against Schistosomiasis

**DOI:** 10.3390/ijms27114799

**Published:** 2026-05-26

**Authors:** Célia Faustino, Lídia Pinheiro, Noélia Duarte

**Affiliations:** iMed.ULisboa, Research Institute for Medicines, Faculdade de Farmácia, Universidade de Lisboa, Avenida Prof. Gama Pinto, 1649-003 Lisbon, Portugal; cfaustino@ff.ulisboa.pt (C.F.); mduarte@ff.ulisboa.pt (N.D.)

**Keywords:** schistosomiasis, terpenes, terpenoids, medicinal plants

## Abstract

Schistosomiasis remains one of the most significant neglected tropical diseases (NTDs) worldwide, sustained by the complex biology of *Schistosoma* species and the host’s immunopathological responses to tissue-trapped eggs. Despite decades of reliance on praziquantel (PZQ) as the sole chemotherapeutic option, major limitations persist, including its lack of activity against juvenile worms, incomplete protection against reinfection, and concerns regarding emerging tolerance. These challenges, together with persistent hotspots of transmission and uneven global progress toward disease elimination, underscore the urgent need for alternative or complementary therapies. Plant-derived terpenes have emerged as promising antischistosomal candidates due to their structural diversity, broad-spectrum bioactivity, and favourable safety profiles. Evidence from in vitro and in vivo studies demonstrates that monoterpenes, sesquiterpenes, diterpenes, triterpenes, and triterpenoid saponins exert multimodal effects on *Schistosoma*, including tegumental disruption, interference with metabolic and redox pathways, inhibition of oviposition, and modulation of host immune and fibrotic responses. Advances in mechanistic studies, supported by omics and computational approaches, further highlight their potential as leads for drug development. Additionally, nano-enabled delivery systems offer strategies to overcome pharmacokinetic limitations and enhance therapeutic performance. This review integrates current knowledge on schistosome biology, treatment challenges, and the growing evidence supporting terpenoids as viable components of a diversified antischistosomal therapeutic arsenal.

## 1. Introduction

Schistosomes are digenetic parasitic blood flukes (or flatworms) of the phylum *Platyhelminthes* and class *Trematoda*. In contrast to the hermaphroditic nature of all other trematode species, schistosomes have separate sexes as adults and cause schistosomiasis by depositing eggs within the vasculature surrounding the host’s intestinal or urinary tract [1,2]. Schistosomiasis, historically referred to as bilharzia, is a debilitating parasitic disease closely associated with poverty and chronic ill health, with transmission occurring in both human and livestock reservoirs. It remains highly prevalent across tropical and subtropical regions and results from infection with one of eight trematode species of the genus *Schistosoma* [3,4,5,6]. The principal species that lead to several types of diseases are *Schistosoma mansoni* (hepatic/intestinal), *Schistosoma haematobium* (genitourinary), and *Schistosoma japonicum* (arteriovenous), whereas *Schistosoma guineensis* (rectal), *Schistosoma intercalatum* (rectal), *S. mattheei* (urinary), *S. malayensis* (arteriovenous), and *Schistosoma mekongi* (arteriovenous) contribute to a smaller global burden of infection (Table 1). In addition to the species that infect humans, *S. bovis*, *S. hippopotami*, *S. curassoni*, *S. rahhaini*, *S. spindale*, *and S. indicum* have been identified as causative agents of schistosomiasis in animal hosts [4,7].

Apart from socioeconomic determinants, such as poverty, migration, urbanization, population displacement, and climate change, schistosomiasis transmission largely depends on environmental conditions influencing the intermediate snail host, particularly proximity to infested water bodies. Moreover, ecotourism and travel to remote regions have led to a growing number of schistosomiasis cases among tourists, who may at times present with severe acute manifestations or atypical complications, including neurological involvement such as paralysis.

The disease is formally categorized among the neglected tropical diseases (NTDs) and is surpassed only by malaria in the magnitude of its global infection burden and in the size of the at-risk population [3,4,8].

According to the 2021 Global Burden of Disease (GBD) analyses, schistosomiasis is currently documented in more than 78 endemic countries, placing nearly 800 million individuals at risk of infection. In this regard, *S. haematobium* transmission was recently confirmed in Cabo Verde—a nation not previously classified as endemic. Globally, the disease accounts for an estimated 70 million disability-adjusted life years (DALYs), underscoring its substantial and persistent public health impact, where adolescents and young adults, particularly those aged 15–24 years, experience the highest age-standardized prevalence and DALYs associated with the disease. Outlined by the same study, the disease remains markedly uneven in its geographical distribution: while the American and Asian continents show burdens that fall well below the global mean, Africa continues to bear a disproportionately high share of the worldwide disease impact, with 93.9% of individuals requiring treatment, where school-aged children (SAC) are highly vulnerable, frequently affected by high-intensity infections [5,6,9,10]. Recent estimates indicate that at least 253.7 million individuals required preventive treatment for schistosomiasis in 2024. Such treatment, which must be administered repeatedly over several years, plays a critical role in reducing and preventing disease-associated morbidity [10]. Despite this broad geographic distribution, large-scale preventive chemotherapy, in which entire populations or communities receive treatment, is required in only 50 endemic countries with moderate to high levels of transmission.

Long-term forecasting analyses reinforce the expectation of a sustained global decline in the burden of schistosomiasis over the coming decades, although with marked regional heterogeneity. Projections based on GBD 2021 data suggest that by 2030, several regions—including the Americas and much of Asia—are likely to meet the WHO threshold for elimination as a public health problem, whereas the African continent is expected to lag despite continued reductions in morbidity and mortality [5,9]. Extending this horizon, bearing in mind some limitations, the Bayesian Age Period Cohort (BAPC) model used by Li et al. [5] predicts a continued downward trajectory in global prevalence, DALYs and mortality through 2041, with Africa consistently remaining the region of highest burden. Complementing these findings, Shen et al. [6] employed Differential Autoregressive Integrated Moving Average (ARIMA) and Exponential Smoothing (ES) models and forecast trends up to 2046, anticipating further declines in female mortality and overall disease burden, although male mortality rates appear more stable. Despite the overall positive trajectory, their analysis underscores that improvements will remain uneven, with low Socio-Demographic Index (SDI) and African regions continuing to bear a disproportionate share of the global burden.

Current control strategies emphasize periodic, targeted administration of praziquantel (PZQ), which remains the first-line therapy for all human schistosomiasis (including the use in preventive chemotherapy campaigns), through large-scale treatment of at-risk groups, complemented by access to safe water, improved sanitation, health education, and surveillance [3,10].

Although both safe and highly effective against the adult stages of all *Schistosoma* species that infect humans, this oral antischistosomal drug neither confers protection against reinfection nor eliminates concerns regarding the potential emergence of drug resistance. PZQ lacks activity against early migrating schistosomula, leaving a substantial window of vulnerability during which treatment does not interrupt ongoing transmission [3,4,11]. These shortcomings are reflected in epidemiological data: despite more than 20 years of mass drug administration (MDA) and the distribution of up to 250 million tablets annually, persistent hotspots and areas of disease resurgence remain common, particularly in sub-Saharan Africa. Systematic reviews also highlight dosing challenges in adults, where standard height-based dosing tools often result in suboptimal PZQ administration, thereby reducing therapeutic efficacy and potentially contributing to selection pressure on parasite populations [11,12]. Several drugs, belonging to distinct chemical classes characterized by sulphur-containing heterocycles, phosphorus-based structures, or nitro groups (such as oxamniquine), were used as alternative therapies to PZQ [4,13,14], being abandoned due to their higher toxicity or lesser activity.

Although ongoing efforts in preclinical development and several vaccine and drug trials offer cautious optimism, progress in antischistosomal drug discovery remains slow. Notably, no new antischistosomal compounds are currently in clinical trials. A detailed understanding of praziquantel’s mode of action and emerging resistance mechanisms is therefore essential to guide the rational design of effective next-generation therapies [2,11].

Given the aforementioned constraints, antischisostomal compounds from plant sources have gained increasing attention due to the urgent need to diversify the drug pipeline [4,11,14,15]. Numerous phytochemicals exhibit antiparasitic activity, and among them, terpenes represent one of the most compelling classes of natural products in the search for novel antiparasitic agents, owing to their remarkable structural variability, broad-spectrum bioactivity, and generally favourable safety profiles. Their ability to exert multimodal antiparasitic effects—ranging from inducing parasite cell death and disrupting stress-response pathways to inducing morphological and ultrastructural alterations and modulating host immune and inflammatory responses—underscores their therapeutic versatility and translational potential. Recent evidence highlights several terpenoid scaffolds with potent activity against key NTDs, including leishmaniasis, schistosomiasis, and echinococcosis, reinforcing the view that terpenes constitute a rich and underexploited reservoir for the rational development of next-generation antiparasitic drugs [4,16].

Schistosomiasis is a major parasitic disease sustained by a highly adapted and complex life cycle, and by the host’s inflammatory response to tissue-trapped eggs. The resulting pathology underpins the chronic morbidity associated with infection, while treatment remains dependent on PZQ alone. In this review, the schistosome life cycle and pathogenesis are initially outlined, since a concise understanding of schistosome biology and of the immune processes driving disease progression is essential to frame present therapeutic challenges. The current therapeutic landscape of schistosomiasis and the persistent challenges that hinder effective long-term control are then discussed, followed by a detailed examination of the growing body of evidence on plant-derived terpenoids—including monoterpenes, sesquiterpenes, diterpenes, triterpenes, and triterpenoid saponins—highlighting their demonstrated antischistosomal properties and their ability to modulate key host immune pathways. By integrating both antiparasitic and immunomodulatory perspectives, we aim to clarify the translational potential of these natural compounds as complementary or alternative strategies to PZQ within a future, more diversified therapeutic arsenal.

The literature search was performed between October and November 2025 using the PubMed, Web of Science, and ScienceDirect databases. A structured combination of keywords, with appropriate truncation adapted to each database, was applied (e.g., combinations of “schistosomiasis” with “terpenes”, “terpenoids”, and “medicinal plants”). Only peer-reviewed articles published in English within the last ten years (2015–2025) were considered. The retrieved records were independently screened by the authors based on quality, accuracy, and relevance to the scope of this review. Reference management, including duplicate removal, was carried out using Mendeley Reference Manager (2020).

## 2. Schistosomiasis: Biology, Pathology, and Current Treatment Challenges

### 2.1. Biology and Life Cycle of Schistosoma

Human schistosomiasis is caused by several species of *Schistosoma*, each exhibiting distinct morphological features that are crucial for diagnosis, epidemiology, and clinical correlation. The main species infecting humans include *S. haematobium*, *S. mansoni*, *S. japonicum*, *S. mekongi*, and *S. intercalatum*/*S. guineensis* [1,3,4,6,17,18,19]. However, hybrid schistosomes arising from interactions between human-infective *S. haematobium* and livestock-associated species such as *S. bovis*, *S. curassoni*, and *S. mattheei* (implicated in intestinal disease in livestock) represent an emerging concern. Their genetic admixture may generate heritable traits that compromise control tools, including preventive chemotherapy. These hybrids are likely to persist in newly colonized regions provided that suitable intermediate snail hosts are present, often displaying expanded host ranges, increased infectivity, and signs of hybrid vigour, thereby complicating schistosomiasis control and elimination efforts [7,20,21]. Endemic in Malaysia, *S. malayensis* is another zoonotic species that causes intestinal schistosomiasis and circulates predominantly in rodent reservoirs. This species makes control particularly challenging, as effective elimination requires not only reducing transmission in human definitive hosts—where infections have been reported only sporadically, with the most recent case documented in 2011 through post-mortem identification of hepatic granulomas—but also addressing the numerous animal reservoirs that sustain the parasite’s life cycle [1].

Although *S. haematobium* is a major contributor to mortality—most notably through its frequent progression to renal failure—the predominant global burden of schistosomiasis arises from chronic morbidity. With an evolutionary association spanning roughly 200,000 years, *S. mansoni* is highly adapted to establish long-term human infections, with adult worms surviving an estimated 6–10 years. This adaptation results in largely asymptomatic infections in most individuals, although a minority develop severe hepatic pathology, including fibrosis, hepatosplenomegaly, and portal hypertension [17].

*Schistosoma* species biology and structure underpin the patterns of egg deposition and the organ-specific pathology observed in intestinal and urogenital disease. Table 1 presents their geographical distribution, main egg morphological characteristics, adults’ location, and relevant clinical manifestations [1,7,8,22,23,24,25]. Across species, adult schistosomes share a dioecious structure (distinct sexes), unlike most trematodes, with a robust male that continuously houses the female in a gynecophoral canal, and a heptalaminate tegument that enables immune evasion and selective drug uptake, contributing to stage-specific PZQ susceptibility. These structural features are central to their survival and explain the resistance of juvenile worms to praziquantel. Concerning morphology, females are longer, slender, and filamentous with higher intravascular motility, whereas males are shorter and more robust, whitish in appearance, and dorsoventrally flattened. By folding their ventral margins inward, males form the gynecophoral canal that encloses the female.

Reported typical adult dimensions range from (6–15) mm × (0.5–1) mm for males, and (10–26) mm × (0.25–0.4) mm for females, depending on strain, age, host, and measurement method [22,25]. Among human schistosomes, *S. mansoni* is the smallest species and *S. japonicum* the largest. Both sexes possess oral and ventral suckers, the latter used for attachment to the endothelium. Schistosomes feed on host blood, and haemoglobin digestion within the gut imparts a dark pigmentation, particularly evident in females, which ingest erythrocytes at substantially higher rates to meet the higher metabolic demands of continuous egg laying [26,27]. Within the gut, hemoglobin from lysed erythrocytes is degraded and its nutrients, including heme-derived iron, are mobilized to support tissue metabolism and eggshell formation.

Human schistosomiasis is not endemic in Europe; however, sporadic autochthonous transmission of *S. haematobium* has been reported in Portugal, Spain, France, Italy, Greece, and Cyprus, reflecting the introduction of the parasite into freshwater bodies harbouring susceptible *Bulinus* or *Planorbarius* snails [18,28]. Intensifying migration, the wide distribution of competent snail hosts, climate-driven expansion of gastropod habitats, and anthropogenic dispersal mechanisms increase the likelihood of further introductions. Although transmission of *S. mansoni* has not been documented, invasive *Biomphalaria* species capable of sustaining this parasite have already been detected in parts of Europe. Avian schistosomiasis, by contrast, is widespread throughout Europe (e.g., Czechia, Denmark, France, Germany, Iceland, Poland, Spain, and Switzerland) and globally (e.g., Argentina, Australia, Canada, Chile, Iran, Japan, the Netherlands, New Zealand, Saudi Arabia, South Africa, and the USA), with aquatic birds as its natural hosts. Humans may act as accidental hosts when avian cercariae penetrate the skin, but these larvae die within the dermis and elicit a localized hypersensitivity response known as cercarial dermatitis or swimmer’s itch. Although generally mild and often underreported, cases are common in summer, especially among individuals with frequent exposure to freshwater [18]. Overall, the burden of schistosomiasis in Europe is probably underestimated, owing to mild clinical presentations, limited surveillance, and low-intensity outbreaks that go undetected.

**Figure 1:**
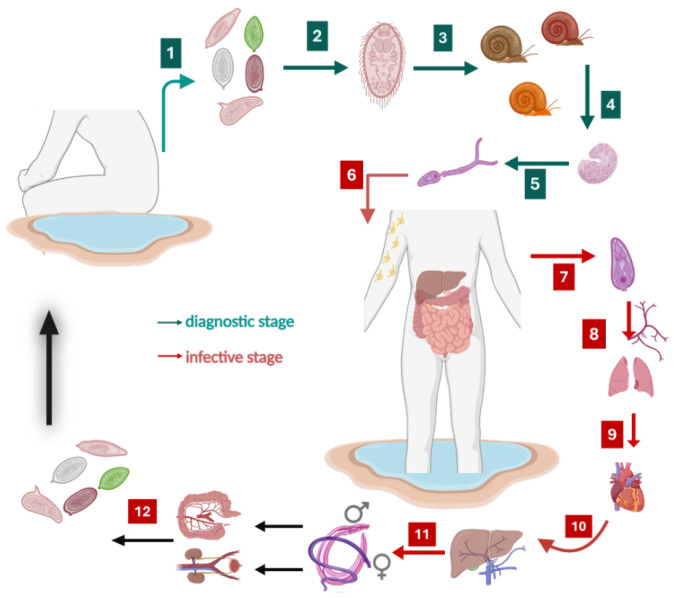
Life cycle of *Schistosoma* parasite: **1**. eggs shed in feces or urine into fresh water; **2**. miracidial release upon egg hatching; **3**. miracidia penetration into specific intermediate hosts (snails); **4**. development of subsequent sporocyst generations; **5**. release of cercariae into water; **6**. penetration of cercariae in human host skin; **7**. tail shedding and Schistosomulae formation; **8**. Schistosomulae migration to lungs via venous circulation; **9**. Schistosomulae migration to left heart and into circulation; **10**. Schistosomulae maturation in the liver; **11**. sexual pairing in host’s venous system; **12**. egg production. Created in https://BioRender.com.

All 23 schistosome species (humans and non-humans) that are currently recognized share the fundamental features of the schistosome life cycle. Schistosome worms, which depend on narrowly defined environmental conditions for their survival, require two distinct hosts to complete their complex reproductive cycle, which involves both asexual and sexual phases (Figure 1). Adult schistosomes reside in human or animal definitive hosts, while a compulsory asexual stage occurs within specific freshwater snail species [17].

Transmission occurs in settings lacking adequate sanitation, when infected human excreta (specifically feces and urine, but occasionally bodily secretions such as semen and menstrual blood) frequently enter natural freshwater, thereby releasing parasite eggs into the environment. Agricultural practices can further amplify transmission; in certain regions of the world, the use of human feces as fertilizer in irrigated fields disperses schistosome eggs across large areas, facilitating their hatching and subsequent infection of suitable snail intermediate hosts.

Eggs are large, oval, and equipped with a lateral or terminal spine whose morphology permits species identification. Asian species produce smaller, rounder eggs with a reduced spine. Daily egg output ranges from 250 to 400 in *S. mansoni*, 200 to 1000 in *S. haematobium*, and 1500 to 3500 in *S. japonicum*. The eggs of *S. haematobium* possess a characteristic terminal spine that functions as a cutting apparatus [27].

After schistosome eggs hatch in freshwater, free-swimming miracidia are released. Induced by osmotic shock, these short-lived ciliated larvae, whose dense covering of surface cilia enables rapid locomotion (approximately 2 mm/s), allowing them to traverse substantial distances in the aquatic environment, must locate and penetrate a compatible snail host within a few hours. Guided partly by snail-derived, genus-specific chemotactic signals, miracidia must infect a suitable intermediate host within roughly 24 h, a survival window modulated by water temperature, salinity, and light [4,8,27].

Among the human-infective species, *S. haematobium* utilizes *Bulinus* spp. snails as its intermediate host; *S. mansoni* depends exclusively on freshwater *Biomphalaria* spp. snails; *S. japonicum* requires amphibious *Oncomelania* spp. snails to sustain its intermediate developmental stages, and *S. mekongi*, *S. malayensis*, and *S. intercalatum*/*S. guineensis* infect snails of the genus *Neotricula* spp., *Robertsiella* spp. and *Bulinus* spp. snails, respectively [4,8,18].

After entering through the snail’s foot, miracidia differentiate into tubular mother sporocysts (typically located within the host’s fibromuscular tissue, most commonly at the site of parasite entry) and undergo clonal asexual development, producing multiple generations of secondary sporocysts that proliferate through snail tissues. This stage involves extensive gene expression changes that enable adaptation to the snail host.

In optimal intermediate hosts, this amplification system generates large numbers of furcocercariae (the free-swimming Schistosoma larval stage), which eventually emerge into the water as the infective stage. The developmental process is strongly temperature-dependent and is also influenced by pH and turbidity. Under these environmental conditions, it unfolds over several weeks in African schistosomes (*S. mansoni*, *S. haematobium*) and over several months in Asian schistosomes (*S. japonicum*) [8,27,29,30].

In human-infecting schistosomes, furcocercariae, which possess a body and a long, forked tail, measuring 400–600 μm, are released from the snail in response to bright light [4]. The light stimuli enhance host-finding efficiency despite their short free-living lifespan of about two days. Each infected snail may release several hundred cercariae per day. These larvae may be transported over long distances by water currents and can penetrate the intact skin of a compatible mammalian host, including humans or cattle, as they are attracted to hydrocarbons and fatty acids present on the host’s epidermal surface. Penetration can trigger a transient allergic dermatitis, which also occurs when non-human schistosome cercariae enter the skin (swimmer’s itch) but subsequently die.

After skin penetration, furcocercariae shed their bifurcated tails and glycocalyx to become the subsequent larval stage named schistosomula, forming their double-membrane tegument within hours [8,17,27]. Once they traverse the skin, schistosomula require two to three days to reach the underlying vasculature and enter the systemic circulation, initiating their migratory journey through the host. Their first major destination is the lungs (within 5–7 days), through the right heart, where high parasite burdens may elicit a transient pulmonary reaction, often characterized by a dry, harsh cough, and before reaching the left heart and progressing to the liver [3,17,29].

The heptalaminate tegument, a syncytial, metabolically active surface with a double lipid-bilayer appearance that lies in direct contact with host blood and facilitates microvesicle-mediated signalling, is a distinctive feature of adult schistosomes. This specialized interface enables immune modulation/subversion and selective drug uptake, underpinning the relative praziquantel tolerance of immature worms and the susceptibility of adults. Moreover, the tegument’s rapid turnover, capacity for epitope rotation/reconfiguration, and self-repair impede durable antigen exposure, posing major challenges to the development of protective or transmission-blocking vaccines [8].

In the hepatoportal system, and after encountering a partner of the opposite sex, they mature into adult male or female worms (measuring about 1 cm in length), which later relocate to mesenteric or pelvic venous plexuses to lay eggs. This developmental sequence takes approximately 40 days, and adults typically survive 3–5 years, occasionally far longer [8,17,27,29].

Adult schistosomes, which exhibit pronounced sexual dimorphism, reside as male–female pairs within the lumen of blood vessels. The male possesses a longitudinal ventral groove, the gynaecophoral canal, an adaptive structure that houses and supports the slender female throughout adult life, ensuring continuous pairing, facilitating her growth and sexual maturation, and enabling sustained egg production. Depending on the species, these paired adults localise to the mesenteric and intestinal venous plexuses (*S. mansoni*, *S. intercalatum*, *S. guineensis*, *S. japonicum*, *S. mekongi*) or to the pelvic and urogenital venous plexuses (*S. haematobium*).

Females deposit their eggs within capillary walls, from which the fertilised eggs may either enter the bloodstream or traverse the intestinal epithelium to reach the vascular lumen, where many become trapped in small terminal venules. In infections caused by *S. haematobium*, mature females deposit eggs within the venous network of the bladder, and they subsequently traverse the bladder wall and are eliminated in the urine, thereby sustaining the transmission cycle [29].

During maturation, schistosomes incorporate host-derived antigens into their tegument through molecular mimicry, a strategy that enables them to evade immune detection and persist within the host for years [4,27,29,30]. Because they are metabolically active and highly immunogenic, the eggs (encapsulated by vascular endothelium cells) trigger immune-mediated reactions that lead to granuloma formation composed of immune cells, fibroblasts, and collagen. This mechanism promotes their translocation across tissues into the bladder, ureters, or intestines, from which they are ultimately excreted in urine or stool, pursuing the cycle [27,29,30].

Although many eggs are transported by the bloodstream to sites that permit their elimination (e.g., *S. mansoni* through the intestine and feces, *S. haematobium* via the bladder and urine), a substantial fraction becomes sequestered within host tissues. Their retention provokes chronic inflammation, which promotes fibrosis, calcification, and may ultimately predispose affected individuals to bladder cancer (*S. haematobium*) or to colorectal cancer [29,31].

Recent work supports the long-standing hypothesis that schistosome development is sustained by totipotent stem-cell lineages known as germinal cells. Histological, molecular, and functional genomic analyses have revealed multiple stem-cell populations in *S. mansoni*, each distinguished by specific gene-expression profiles. Some larval stem cells give rise to the adult stem cells that maintain growth and tissue renewal, whereas others specify the germline lineage responsible for gamete formation [32].

### 2.2. Pathogenesis and Host Immune Responses

Clinical schistosomiasis evolves through three overlapping phases: acute infection, established active disease, and late chronic pathology, each reflecting the duration and progression of infection [3]. These stages differ in characteristic symptomatology and in the patterns and intensity of egg excretion in stool or urine.

#### 2.2.1. Acute Infection

Acute schistosomiasis typically occurs in humans following primary exposure and cercarial penetration of intact skin in freshwater of endemic areas, where some larvae die locally while others enter the venous or lymphatic circulation and subsequently reach the liver for maturation. In the skin, innate immune responses to degenerating larvae can elicit a pruritic maculopapular hypersensitivity reaction related to the innate immune activation cycle [3,8,17].

After cercarial penetration and schistosomula maturation, the infection may progress to an acute symptomatic phase. This presentation—commonly referred to as acute schistosomiasis, often termed as Katayama syndrome or Katayama fever—typically arises in previously unexposed individuals within a few weeks to three months after first exposure. Clinical manifestations result from systemic hypersensitivity responses and immune complex formation triggered by antigens released during larval migration or the onset of egg deposition. They are frequently accompanied by eosinophilia and transient pulmonary infiltrates. The illness is characterized by fever, right-upper-quadrant abdominal pain, malaise, fatigue, myalgia, abdominal pain, diarrhoea (with or without blood); haematuria may accompany *S. haematobium infection* [3,8].Although the full Katayama syndrome is most often associated with *S. mansoni* and *S. japonicum*, with a greater severity for the latter, early manifestations can occur with other human schistosomes; nonetheless, the acuity and prominence of early systemic symptoms vary across species and host factors. In endemic regions for *S. mansoni* or *S. haematobium*, acute schistosomiasis is seldom observed among residents; the observed reduced susceptibility may reflect in utero desensitization, which dampens infant immune responses to schistosome antigens, or repeated exposure to skin-penetrating cercariae, which promotes IL-10-mediated regulatory responses by cutaneous CD4^+^ T cells [3].

#### 2.2.2. Established Active Infection

In most individuals, particularly those residing in endemic regions, acute symptomatic disease does not occur, and the infection progresses directly to an established active phase, characterized by mature adult worms and consistent egg production; during this stage, viable eggs are excreted in stool or urine. Adult worms within the vasculature elicit little or no local inflammation, owing to their ability to continually renew their tegument through somatic stem cells and to mask surface antigens by adsorbing host molecules [3].

The principal clinical manifestations and organ-specific lesions of patent infection arise from immune responses to parasite eggs. Schistosome eggs actively secrete antigenic glycoproteins that promote their passage from the vasculature into the intestinal or urinary lumen by inducing localized inflammation. However, these secreted antigens also drive granuloma formation around eggs that become trapped in tissues, with granulomas composed predominantly of eosinophils, neutrophils, lymphocytes, and macrophages [33].

Organ-specific manifestations generally scale with infection intensity and result from egg-driven inflammation and granuloma formation. In endemic settings, established active schistosomiasis—common in children—is fully reversible after clearance of adult worms. The robust lymphocyte responses to soluble egg antigens (SEAs) at this stage progressively diminish as the infection transitions to chronicity [33].

#### 2.2.3. Late Chronic Pathology

In individuals with continuous exposure in endemic areas, worm burdens typically decline after early adolescence as partial immunity to reinfection develops and existing adult worms die naturally. Consequently, egg excretion and tissue deposition decrease. Concurrently, new granulomas become smaller owing to immunological downregulation, while older granulomas resolve as their enclosed eggs degenerate, leaving behind fibrotic tissue. This remodeling process contributes to a gradual reduction in symptom severity. Over time, the chronic responses lead to the accumulation of innumerable microscopic scars, progressive fibrosis, and eventual obstruction or dysfunction of the affected organs, demonstrating that the pathology of schistosomiasis arises predominantly from the host’s inflammatory response to retained eggs. Consequently, portal hypertension, gastrointestinal bleeding, hepatosplenomegaly, hepatic encephalopathy, and ultimately liver failure may occur [8,27].

Late chronic infection with *S. mansoni*, *S. mekongi*, *S. japonicum*, *S. guineensis*, and *S. intercalatum*, all of which inhabit the mesenteric venous plexus, leads to intestinal schistosomiasis. Chronic intestinal schistosomiasis causes abdominal pain and diarrhoea and may advance to periportal fibrosis, portal hypertension, splenomegaly, and variceal bleeding, particularly in *S. mansoni* and *S. japonicum* infections, reflecting their mesenteric venous distribution. As the disease progresses, it may extend to involve the liver and spleen, culminating in the development of hepatosplenic schistosomiasis. On the other hand, the late chronic infection with *S. haematobium*, which resides in the pelvic venous plexus, results in urogenital schistosomiasis, predominantly affecting the bladder wall; these clinical consequences lead to haematuria, dysuria, and fibrosis-related urinary tract obstruction, with an elevated risk of bladder squamous-cell carcinoma [8,27]. Female genital schistosomiasis, a recognized consequence of *S. haematobium* infection, involves lower-genital-tract lesions and increases vulnerability to adverse reproductive outcomes; moreover, urogenital schistosomiasis has also been implicated as a co-factor associated with increased risk of HIV transmission [3,8]. Morbidity is greatest in high-intensity infections, particularly those due to *S. mansoni* and *S. japonicum*. In chronic schistosomiasis, particularly in endemic settings, the disease is frequently associated with undernutrition, reduced physical performance, diarrhoea, abdominal discomfort, and anaemia, reflecting the cumulative effects of persistent egg-induced inflammation and chronic blood and nutrient loss.

Although most clinical manifestations involve the genitourinary, intestinal, or hepatosplenic systems, schistosomiasis can occasionally affect other organs when adult worms migrate and deposit eggs ectopically. Autopsy studies have identified worms and eggs in nearly every organ, reinforcing that schistosomiasis may be systemic rather than strictly localized. Clinically, however, pulmonary and neuroschistosomiasis remain the most relevant ectopic presentations [8].

#### 2.2.4. Parasite-Driven Immunomodulation in Schistosomiasis

The interplay between the host immune system and schistosomes spans multiple parasite stages, including penetrating cercariae, migrating schistosomula, adult worms, and tissue-trapped eggs, and underlies both protective and pathological processes. Immune responses directed against larval stages contribute to the gradual development of resistance to reinfection, whereas reactions to egg antigens drive the characteristic immunopathology of schistosomiasis. Protective immunity emerges slowly over 10–15 years, explaining why young children in endemic areas remain highly susceptible to reinfection after treatment, while adults exhibit increasing resistance. This age-dependent pattern parallels epidemiological observations of peak prevalence and intensity in childhood [3].

Considering the immune response to *S. mansoni*, the migration of schistosomula through host tissues elicits a predominantly type-1 immune response during the first several weeks of infection. This early phase is marked by heightened production of interleukin-12 (IL-12) and interferon-γ (IFN-γ), reflecting a response largely directed against larval and early worm antigens. Such Th1-type response persists until approximately five weeks post-infection, coinciding with the period before the onset of egg deposition and the subsequent immunological shift driven by the maturing parasite [17,33].

As the parasite matures and begins to lay eggs, typically around five to six weeks post-infection, the host immune response undergoes a pronounced shift from a type-1–dominated profile to a type-2–oriented one, where eggs are the principal drivers of this immunological reprogramming. This transition is marked by reduced IFN-γ production and the polarization of CD4^+^ T helper cells toward a Th2 phenotype.

The most profound immunomodulatory events occur after oviposition. The protective type-2 immune response is driven by the parasites’ SEAs and includes the expansion of Th2 lymphocytes, eosinophils, and basophils; enhanced production of IL-4, IL-5, and IL-13; class-switching towards IgG1 and IgE; and the M2 macrophage polarization. As infection progresses into the chronic phase (beyond three months), the intensity of this Th2-driven response gradually wanes, accompanied by a decline in granulomatous inflammation around tissue-trapped eggs. During this period, regulatory T and B cells, and anti-inflammatory cytokines (e.g., IL-10, TGF-β) expand, promoting a state of immune hyporesponsiveness that modulates pathology while permitting long-term host–parasite coexistence [17,33]. Modern proteomic and transcriptomic studies reveal that SEAs include glycoproteins, proteases, and molecules with TLR-modulating properties that suppress dendritic cell maturation and skew macrophages toward an anti-inflammatory phenotype [34]. These SEA-driven programs ensure a sustained environment of immune deviation, tolerance, and wound-healing-type responses that benefit the parasite during chronic infection and reinforce long-term survival.

It is interesting to note that the egg-induced granuloma fulfils several critical functions for both host and parasite. Intestinal granulomatous inflammation facilitates the translocation of eggs into the gastrointestinal lumen, ameliorating bacterial translocation from the intestine into the systemic circulation. In addition, it protects host tissues from excessive immune activation directed against highly antigenic eggs, and it ultimately preserves host integrity, thereby supporting long-term survival of the adult worms [17]. Recent analyses emphasize that the Th2 response is a paradoxical one: protective in the short term, yet pathogenic when sustained chronically, contributing to hepatic and intestinal fibrosis [35].

Successful translocation of *S. mansoni* eggs to the intestinal lumen is far from assured. Approximately half of all eggs laid by adult worms fail to reach the gut and are instead carried to the liver, where they become lodged in the sinusoids and elicit intense granulomatous inflammation [2,17].

Together, the acute Th2 polarization and the regulatory milieu of chronic infection create the immunological landscape that schistosomes exploit to ensure that their eggs traverse host tissues efficiently. Growing insight into schistosome immunobiology has made it clear that *Schistosoma* species deploy multiple strategies to promote efficient egg transit. Within the vasculature, angiogenesis, endothelial activation, and interactions with coagulation components facilitate egg extravasation. In intestinal tissues, schistosomes shape local immunity to favour granuloma formation around transiting eggs—an essential prerequisite for successful excretion. To prevent excessive pathology during chronic infection, they further steer the host response toward a more regulatory phenotype.

Hepatic egg granulomas are well characterized and, unlike intestinal granulomas, cannot be expelled and progressively become fibrotic. Histological analyses show that most productive, collagen-rich granulomas in *S. mansoni* infection arise in the liver, whereas intestinal granulomas appear more organized and contain fewer circumferential collagen fibers. Hepatic granulomas decrease in size between weeks 8 and 20 post-infection, reflecting the development of chronic immune hyporesponsiveness, while angiogenesis contributes to portal fibrosis and tissue remodeling. Importantly, liver granulomas also protect the host by sequestering hepatotoxic egg secretions, particularly omega-1 (a hepatotoxic egg glycoprotein).

PZQ treatment partially reverses this regulatory imprinting by reducing worm burden and lifting parasite-induced immunosuppression. Post-treatment immune shifts include increases in Th1 and Th17 responses, reductions in FOXP3^+^ Tregs, normalization of T-cell subsets, and enhanced generation of parasite-specific antibodies. However, these changes are neither uniform nor durable: reinfection rapidly re-establishes Th2/regulatory polarization, while elements of long-term immunological imprinting—particularly myeloid and stromal reprogramming—persist despite parasite clearance [36]. In endemic settings, PZQ treatment has been leading to a temporary uncloaking of the Th1/Th17 axis. However, reinfection reinstates the Th2/Treg state, leading to immune oscillation cycles that shape a unique collective immunological phenotype [37].

### 2.3. Current Treatment Landscape and Emerging Challenges

#### 2.3.1. Praziquantel

**Figure 2:**
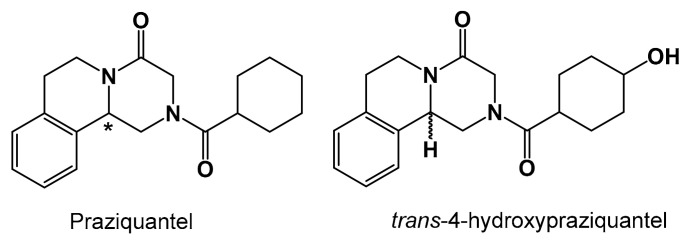
Praziquantel (PZQ) and its main metabolite, *trans*-4-hydroxy-praziquantel (4-OH-PZQ). The asterisk (*) in the chemical structure of praziquantel indicates the chiral center of the molecule (the asymmetric C-11b carbon).

Praziquantel (PZQ; Figure 2) remains the drug of choice for the treatment of all forms of schistosomiasis due to its efficacy, good safety profile, low cost, and the lack of alternative drugs. PZQ is a pyrazino-isoquinoline derivative developed in the 1970s with anthelminthic activity against different cestodes and trematodes, including schistosomes, whereas nematodes are unaffected [38,39]. Schistosomes exhibit stage-dependent as well as sex-specific and pairing status-dependent differences in PZQ susceptibility, with male worms showing higher sensitivity than females, likely because females residing within the male’s gynecophoral canal experience partial shielding from drug exposure [40,41]. The major drawback of PZQ is its low efficacy against juvenile schistosomes, with re-infections being frequent.

PZQ is effective against all human schistosome species and their various clinical manifestations, including advanced hepatosplenic disease [38,41]. The drug crosses the blood–brain barrier (BBB), which allows treatment of *S. japonicum*-induced cerebral schistosomiasis and neurological syndromes caused by *S. mansoni* and *S. haematobium*, sometimes in association with corticosteroids when inflammation is significant [38]. The WHO recommends single-dose treatment with PZQ (40 mg/kg) as preventive chemotherapy for schistosomiasis, and mass drug administration (MDA) strongly reduced morbidity, prevalence, and infection intensity by repeatedly clearing adult worms and interrupting ongoing transmission in exposed communities [42]. PZQ is safe and well-tolerated in children and adults, with usually mild and transient side effects that most often include nausea, anorexia, vomiting, abdominal pain, diarrhea, headache, dizziness, fever, and pruritus [38,41]. More severe side effects, such as bloody diarrhea and edematous urticaria, correlate with the intensity of infection and have been attributed to the release of antigens by dying worms [38,41].

PZQ is usually available in the form of oral tablets (600 mg) as a racemic mixture of the pharmacologically active levorotatory (*R*)-enantiomer (R-PZQ or L-PZQ) and its dextrorotatory (*S*)-diasteroisomer (S-PZQ or D-PZQ), which contributes to the bitter and unpleasant taste of the drug [39,43]. Hence, an orally dispersible tablet (ODT) pediatric formulation of enantiomerically pure R-PZQ (arpraziquantel) to improve palatability and children compliance has been developed, which successfully completed phase 3 clinical trials in preschool-aged children with schistosomiasis [44].

PZQ is readily absorbed (>80%) following oral administration, with peak plasma levels occurring within 1–3 h after dosing [45,46]. However, the drug suffers extensive first-pass hepatic metabolism, mostly hydroxylation to *trans*-4-hydroxy-praziquantel (*trans*-4-OH-PZQ; Figure 2), the main metabolite, which limits its bioavailability. PZQ undergoes rapid clearance from systemic circulation, with more than 70% of an oral dose eliminated within 24 h, mainly in the urine, as inactive hydroxylated and conjugated metabolites [41,46]. PZQ is highly protein-bound (~80% to albumin) and has a plasma half-life usually in the range between 0.8 and 1.5 h, which can be prolonged in patients with hepatosplenic schistosomiasis or liver-compromising diseases [45,46,47].

Despite being on the market for almost 50 years, the mechanism(s) of action and molecular target(s) of PZQ remain elusive. PZQ induces schistosome muscular contraction and spastic (tetanic) paralysis, leading to detachment of affected worms from host tissues and a rapid shift of adult worms from the mesenteric veins to the liver [39,41]. However, clinical efficacy correlates more strongly with the extent and severity of tegumental damage caused by PZQ [48]. Membrane damage exposes parasite’s antigens on the tegument surface, triggering a host immune response that contributes to PZQ effectiveness [49,50].

Membranes of susceptible helminths appear to be the primary target of PZQ, and the drug is known to cause increased membrane permeability to calcium ions, presumably by modulating voltage-gated calcium channels [51,52], which was however contradicted by the lack of correlation between PZQ-induced calcium influx into the schistosome and parasite death [53]. Recently, a Ca^2+^-permeable transient receptor potential (TRP) ion channel of the melastatin subfamily expressed in *S. mansoni* (*Sm*.TRPM_PZQ_) and other PZQ-sensitive flatworms was identified as a target of PZQ, which may help elucidate how the drug perturbs schistosome calcium homeostasis [54,55,56]. Stereoselective activation of *Sm*.TRPM_PZQ_ by R-PZQ engaging with a hydrophobic ligand-binding pocket triggers a rapid influx of calcium ions that drives membrane depolarization, sustained muscle contraction, spastic paralysis, and tegument disruption, ultimately leading to parasite death [55,56].

Despite its effectiveness, monotherapy and MDA in chemoprophylaxis campaigns have raised concerns about the emergence of PZQ resistance. Laboratory-induced resistance of PZQ has been achieved [57,58], and decreased susceptibility of schistosomes to PZQ in the field has been reported following continuous drug exposure [59,60,61], although no robust evidence of the development of PZQ resistance has been found [15,62,63,64]. This growing threat has prompted interest in alternative and cost-effective approaches, notably drug repositioning, with artemisinin-based antimalarial drugs emerging as promising candidates for schistosomiasis chemotherapy, exploiting the heme-rich environments created during host hemoglobin digestion in *Plasmodium* food vacuoles and *Schistosoma* gut lumen [65,66,67,68,69].

#### 2.3.2. Drug Repurposing for Schistosomiasis: Antimalarial Sesquiterpene Lactones

**Figure 3:**
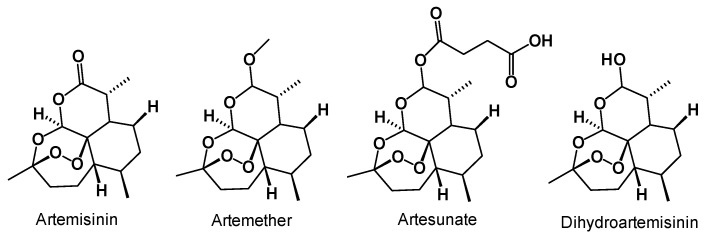
Artemisinin (ART) and its semi-synthetic derivatives artemether (AM), artesunate (AS), and dihydroartemisinin (DHA), antimalarial drugs repurposed for schistosomiasis.

Artemisinin (ART) is a natural antimalarial sesquiterpene lactone with a characteristic endoperoxide bridge (1,2,4-trioxane ring) isolated from *Artemisia annua* (Asteraceae). ART and its semi-synthetic derivatives artesunate (AS) and artemether (AM), and their main active metabolite dihydroartemisinin (DHA), collectively known as artemisinins (ARTs; Figure 3), have demonstrated efficacy against *Schistosoma* species, particularly juveniles, both in vitro and in vivo in animal models of prepatent and patent infections [66,69,70,71,72,73,74], including against PZQ non-susceptible isolates [74]. Female worms are usually more susceptible than males, presumably due to their higher heme exposure and turnover, and this increased susceptibility leads to marked reductions in egg burden and a corresponding decrease in egg-associated granulomatous pathology in the host liver [70,71,72,75]. The major drawback of ARTs is their short half-lives (1–3 h), which requires repeated treatments, and their lower efficacy against adult stages of schistosomes when compared to PZQ [72,76]. Nevertheless, the efficacy and safety of ARTs in the chemotherapy and chemoprophylaxis of schistosomiasis became evident in numerous clinical trials [77,78,79,80,81]. ARTs have been repurposed for schistosomiasis and played a major role in reducing *S. japonicum* transmission in China, where AM and AS were incorporated into treatment, seasonal chemoprophylaxis, and broader elimination programs [72,82].

Although not fully elucidated, the mechanism of action of ART-based antimalarials involves heme-mediated activation of their endoperoxide bridge in the schistosome gut lumen, generating reactive oxygen species (ROS) and carbon-centered radicals [66,69,76,83]. These highly reactive intermediates form adducts with numerous proteins, receptors, and enzymes, including heme itself, disrupting proteostasis, redox balance, metabolism, and antioxidant defenses, while the accumulation of free heme further contributes to oxidative stress [72,76,84].

ART-induced oxidative stress leads to glutathione depletion, lipid peroxidation, and inhibition of several antioxidant and metabolic enzymes [72,85,86,87]. Living in a highly oxidative environment such as the bloodstream, impaired oxidative stress response in the parasite increases susceptibility to ROS, resulting in lesions to the tegument, subtegumental musculature, parenchymal tissues, and degeneration of male and female reproductive organs [72,88,89,90]. Juvenile schistosomes, owing to their high metabolic activity, are particularly vulnerable. Therefore, combination of ARTs and PZQ, with complementary activity against juvenile and adult stages of the parasite, showed improved efficacy in worm and egg reduction rates compared to monotherapy, as demonstrated in experimental animal models of schistosomiasis [84,86,91,92], and several clinical trials [93,94,95,96,97,98]. Most clinical studies focused mainly on combinations of PZQ with AM or AS, and more recently with ART-based combination therapies (ACTs), such as AM-lumefantrine [81], AS-mefloquine [93,97,98], AS-amodiaquine [98] or DHA-piperaquine [96,98], currently the standard treatment for uncomplicated *P. falciparum* malaria, since both diseases are co-endemic in sub-Saharan Africa where co-infection is common [77,78,99,100]. However, in malaria-endemic settings, WHO and national malaria programs prioritize preserving the efficacy of ACTs for *Plasmodium*, thereby avoiding additional selective pressure on parasite populations that could accelerate resistance and undermine malaria-control strategies [99,100].

Systematic reviews and meta-analyses comparing the efficacy of ART-based therapies for the treatment and prophylaxis of human schistosomiasis confirmed that ARTs used in combination with PZQ were associated with increased cure rates in schistosomiasis treatment, but not ART monotherapy, and demonstrated the prophylactic role of ARTs in reducing the incidence of *S. japonicum* infections compared to placebo [101,102]. ARTs were well tolerated and only mild and transient adverse events were observed, most frequently gastrointestinal symptoms (nausea, vomiting, diarrhea and abdominal pain), dizziness, and headache [101,102].

However, the routine use of ARTs in MDA programs, either alone or in combination with PZQ, raises concerns about accelerating artemisinin resistance in malaria parasites in co-endemic regions. Additionally, a gradual decline in the prophylactic efficacy of ARTs-based regimens against *S. japonicum* has been observed in China, where reduced sensitivity to AS was reported after a decade of seasonal chemoprophylaxis [103]. These trends underscore the urgent need to develop alternative cost-effective antischistosomal agents, preferentially active against all human developmental stages of the parasite, and that operate through novel or mechanistically distinct pathways. In this context, terpenes and terpenoids are emerging as promising antischistosomal agents because they possess remarkable structural diversity and a wide range of biological activities, many of which are relevant to schistosome survival [65,104,105,106,107]. Their chemical diversity and distinct mechanisms of action may help overcome or delay drug resistance due to their distinct biochemical targets and lower risk of cross-resistance. Additionally, terpenes are abundant in medicinal plants widely used in endemic regions, making them accessible, affordable, and culturally familiar. Several extracts and essential oils from numerous medicinal plants have been used in traditional medicine worldwide for treatment of schistosomiasis and other parasite-related diseases [104,105,106,107,108,109,110,111,112]. Their potential to act synergistically with PZQ further enhances their relevance, supporting their use in combination therapies aimed at improving efficacy and mitigating the emergence of resistance.

## 3. Terpenes: Classification, Biosynthesis, and Antiparasitic Potential

Terpenes represent the most structurally and functionally diverse class of secondary metabolites, with more than 80,000 naturally occurring compounds identified to date [113]. They are widely distributed across plants, microorganisms, insects, and marine invertebrates, where they play essential roles in primary and secondary metabolism and constitute an abundant source of bioactive molecules [114]. Their remarkable chemical diversity results from the assembly of five-carbon (C_5_) units derived from the precursors isopentenyl diphosphate (IPP) and its isomer dimethylallyl pyrophosphate (DMAPP) [115]. Further extensive rearrangements, cyclization reactions, and functional modifications are catalyzed by prenyltransferases and terpene synthases. Based on the number of isoprene units incorporated, terpenes are conventionally classified as hemiterpenes (C_5_), monoterpenes (C_10_), sesquiterpenes (C_15_), diterpenes (C_20_), triterpenes (C_30_), and tetraterpenes (C_40_). These classes encompass a broad range of structures including linear, bicyclic, tricyclic, and polycyclic scaffolds, many of which serve ecological functions such as defense, communication, and adaptation to environmental stress [114,115].

Numerous terpenes have been reported to exhibit significant antiparasitic activities [116,117]. Monoterpenes and sesquiterpenes—frequently abundant in essential oils—display activity against protozoan parasites such as *Plasmodium*, *Leishmania*, and *Trypanosoma*, often through mechanisms involving membrane disruption, mitochondrial dysfunction, or perturbation of redox homeostasis [116,117]. Diterpenes and triterpenes demonstrate antiparasitic effects, with some compounds acting on key biosynthetic pathways, modulating host–parasite interactions, or exhibiting synergistic effects with established antiparasitic agents [118,119,120]. Given their structural diversity and the multiplicity of potential biological targets, terpenes remain promising candidates for the development of novel antiparasitic drugs, particularly in the context of increasing global resistance to conventional medicines.

## 4. Plant-Derived Terpenes with Antischistosomal Activity

### 4.1. Monoterpenes

Monoterpenes are particularly abundant in the essential oils (EOs) of numerous plants and aromatic herbs. Many are widely used as flavoring agents, fragrance ingredients, preservatives, and skin permeation enhancers in topical formulations, with several being generally recognized as safe (GRAS) by the U.S. Food and Drug Administration (FDA) [121,122]. Owing to their favorable safety profiles [121,123], and broad spectrum of bioactivities, including antimicrobial [124,125,126,127], antioxidant [125,128,129], anti-inflammatory [125,128,129], and antitumoral [129,130,131] effects, monoterpenes represent promising leads in drug discovery.

**Figure 4:**
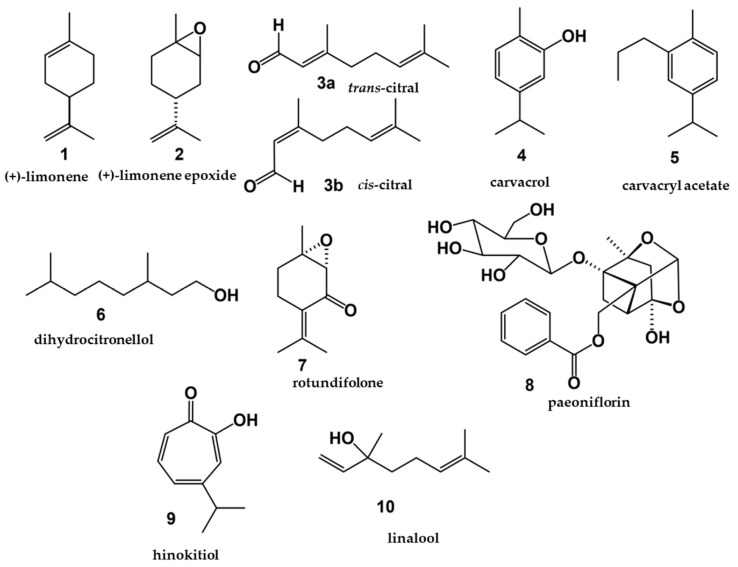
Antischistosomal plant-derived monoterpenoids.

*Citrus* fruits (Rutaceae) produce large amounts of (+)-(*R*)-limonene (**1**; Figure 4), a major constituent of *Citrus* EOs. This monoterpene has demonstrated antibacterial [132,133], antifungal [134,135], and antiprotozoal [136,137,138,139] activities. The compound showed in vitro trypanocidal activity [137,139], leishmanicidal effects in vitro and in vivo [136], and inhibited isoprenoid biosynthesis in *Plasmodium falciparum*, arresting parasite development [137].

Chemical or enzymatic oxidation of the cyclic double bond of (+)-limonene (**1**) may occur in plant tissues, yielding a diastereomeric mixture of *cis*/*trans* isomers of (+)-limonene epoxide (**2**; Figure 4). This mixture exhibited antischistosomal activity comparable to PZQ (10 μg/mL) against 49-day-old adult *S. mansoni* worms in vitro [140]. It caused 100% motility reduction and complete parasite death after 24 h at 75 μg/mL or after 120 h at 25 μg/mL. Confocal laser scanning microscopy revealed that the monoterpenoid’s schistosomicidal activity was associated with tegumental destruction. No significant differences were observed between male and female worms exposed to **2** [140]. Evaluation of acute toxicity of **2** in mice showed an LD_50_ value of 4000 mg/kg following intraperitoneal (i.p.) administration [141]. The compound was well tolerated at i.p. doses up to 1000 mg/kg [141].

Interestingly, the parent compound (+)-limonene (**1**), the major component of *C. limonia* and *C. reticulata* EOs, was inactive against adult *S. mansoni* Luiz Evangelista (LE) strain at concentrations of 12.5–100 μg/mL within 72 h of incubation, despite the antischistosomal activity of the EOs themselves (LC_50_ = 81.7 μg/mL at 24 h) [142]. In contrast, **1** isolated from *Mentha x villosa* decreased the motility of adult *S. mansoni* Belo Horizonte (BH) strain after 96 h at 43.75 μg/mL, which may reflect differences in strain susceptibility and incubation time [143]. Similarly, the phenylpropanoid (*E*)-anethole and **1**, identified as the main components of the leaf essential oil of *Foeniculum vulgare* (fennel), a Mediterranean aromatic plant traditionally used as a vermifuge, showed no activity against *S. mansoni* (LE strain) at 10, 50, or 100 μg/mL within 120 h of incubation [143]. However, binary mixtures of **1** or its enantiomer (−)-(*S*)-limonene with (*E*)-anethole, in the same proportions found in the EO, achieved 100% mortality at 100 μg/mL after 24 h, supporting the idea that synergistic effects are responsible for the moderate antischistosomal activity of the EO, since the individual components were inactive on their own [144].

Among the genus *Lippia* (Verbenaceae), *Lippia alba*, *L. gracilis*, *L. sidoides* and *L. origanoides* EOs and their major terpenes have demonstrated antiprotozoal [137,145,146,147,148] and anthelmintic [149,150,151] activities. A study on the antischistosomal activity of *L. alba* and *L. gracilis* EOs identified citral (**3**) as a mixture of its geometric isomers, *trans*-citral (**3a**) (24.1%) and *cis*-citral (**3b**) (25.9%), known as geranial (**3a**) and neral (**3b**), and limonene (**1**) (18.5%) as the major constituents of *L. alba*, and carvacrol (**4**) (32.3%), γ-terpinene (20.6%), and *p*-cymene (18.95%) as the main components of *L. gracilis* [150]. Carvacrol (**4**) induced 100% mortality of adult *S. mansoni* in vitro after 2 h of incubation at 100 μg/mL, while citral (**3**) reduced worm viability by more than 75% after 24 h of incubation at 50 and 100 μg/mL. However, both compounds showed moderate cytotoxicity toward mammalian cells at these high concentrations, resulting in poor selectivity [150].

Sublethal concentrations (5 μg/mL) of either **3** or **4** significantly reduced oviposition by approximately 90% after 48 h of exposure [150]. This low concentration did not reduce motility or cause separation of adult worms, suggesting that inhibition of oviposition was associated with morphological alterations in the reproductive system of male or female parasites induced by the monoterpenoids. This hypothesis was supported by previous morphological analysis of female *S. mansoni* exposed for 48 h to *L. alba* or *L. gracilis* EOs at sublethal concentrations (5 μg/mL), which showed degeneration of reproductive organs, including vacuolization of the vitelline glands and vitelloduct, reduction of the ovary, and absence of eggs in the ootip [150].

Esterification of carvacrol (**4**) with acetic anhydride, intended to reduce its toxicity and enhance pharmacological activity, yields carvacryl acetate (**5**), a semi-synthetic derivative with an improved acute safety profile (oral LD_50_ > 1500 mg/kg in mice vs. 919 mg/kg for **4**) [152]. Carvacryl acetate (**5**) exhibited in vitro antischistosomal activity against *S. mansoni* at 6.25 μg/mL, reducing parasite motility and viability with no differential sensitivity between male and female worms [153]. At 25 μg/mL, 100% lethality was observed after 24 h. Sublethal concentrations inhibited oviposition without affecting worm pairing, with the highest sublethal dose (6.25 μg/mL) achieving 90% reduction in egg production worms compared with the untreated control group, presumably due to degenerative modifications in the worms’ reproductive system. Confocal laser scanning microscopy revealed tegumental alterations in 49-day-old adult *S. mansoni*, including swollen tubercles and visible blebbing [153].

Further in vitro studies showed that **5** was more effective against *S. mansoni* schistosomula (EC_50_ = 14.58 μM) than against adult worms (EC_50_ = 42.16 μM) [154]. However, in mice with patent *S. mansoni* infection, treatment with a single oral dose of **5** (400 mg/kg) reduced adult worm burden and intestinal and fecal egg loads by 39.2%, 70.1% and 76.6%, respectively, but showed little efficacy in prepatent infection [154]. The low in vivo activity of **5** was partially attributed to its poor pharmacokinetic profile [154].

An in vitro screening of 38 structurally related terpenes against *S. mansoni* identified dihydrocitronellol (**6**; also known as tetrahydrogeraniol), a hydrogenated monoterpene alcohol, as the most active compound (IC_50_ = 13.61 μM at 24 h) [155]. This monoterpene is found in trace amounts in citronella, lemongrass, rose, and geranium oils, but it is usually prepared by hydrogenation of citronellol, citronellal, or geraniol, which are abundant in EOs. The monoterpenoid reduced worm motor activity at concentrations ≥ 10 μM and caused 100% mortality within 24 h at 80 μM or within 120 h at 20 μM [155]. Both male and female adult worms were highly susceptible to its schistosomicidal effects. Confocal laser scanning microscopy showed that **6** produced extensive, concentration-dependent tegumental damage in adult schistosomes, including surface sloughing and tubercle collapse and disruption [155]. Furthermore, a correlation was found between parasite viability and the severity of the tegumental lesions. The amphiphilic nature of **6**, combining a long hydrophobic chain with a terminal hydroxyl group, may facilitate tegumental permeation, while the flexible apolar chain likely enhances interactions with hydrophobic target sites [155]. Acute oral toxicity studies in rats indicate that the compound is practically non-toxic (LD_50_ > 5000 mg/kg) [121].

Rotundifolone (**7**) is a monoterpene ketone abundant in many EOs from *Mentha* species (Lamiaceae), including *M. x villosa*, a plant used in traditional medicine for its antiparasitic activity [156]. An aqueous alcoholic extract of the aerial parts of this plant is commercially available for the treatment of giardiasis, amebiasis, and urogenital trichomoniasis [157]. Rotundifolone (**7**) isolated from the leaf EO of *M. x villosa* showed in vitro schistosomicidal activity against *S. mansoni*, killing 100% of adult worms after 72 h of incubation at 71.0 μg/mL (~0.43 μM) [156,158]. Scanning electron microscopy (SEM) revealed tegumental damage after 24 h of incubation with **7**, including bubble-like lesions and loss of tubercles in the ventral region. After 48 h, destruction of the oral sucker and contraction of the ventral sucker were observed [158]. Oral administration of **7** (141.9 mg/kg/day) for 5 consecutive days to *S. mansoni*-infected mice resulted in a 74.5% reduction in worm burden, which was lower than the 95.9% reduction observed with the positive control PZQ (200 mg/kg) [143]. Less pronounced reduction rates were noted in intestinal, hepatic, and fecal egg counts. Analysis of the oogram pattern showed that **7** decreased the number of immature eggs and increased the number of dead eggs compared with untreated infected mice [143].

Paeoniflorin (**8**) is a water-soluble monoterpene glycoside found in *Paeonia lactiflora* (Paeoniaceae). It is the principal bioactive constituent of peony roots used in traditional herbal medicine in China and other Asian countries, which are used to manage pain, inflammation, immune-related disorders, and liver diseases [159]. The anti-inflammatory and immunomodulatory effects of **8** have been demonstrated [159].

Paeoniflorin (**8**) was investigated for its antiparasitic and antifibrotic effects against *S. mansoni* in a murine model of schistosomiasis, alongside PZQ for comparison [160]. Mice received oral **8** at 50 mg/kg daily or PZQ at 300 mg/kg twice daily for one month, beginning 6 weeks post-infection [160]. Early chemotherapy is recommended, as delayed treatment may not be able to reverse splenomegaly or hepatic fibrosis. The high PZQ dose was chosen based on previous findings showing that low doses (75 mg/kg twice daily) did not improve hepatic fibrosis, likely due to rapid hepatic metabolism [161].

Treatment with **8** was accompanied by significant reductions in hepatic worm burden (84.3% vs. 92.8% for PZQ), immature eggs (95.8% vs. 100% for PZQ), and mature eggs (90.2% vs. 92.9% for PZQ), while increasing the number of dead eggs (18.9-fold vs. 19.6-fold for PZQ) compared with the infected, non-treated group [160]. Although less potent than PZQ at improving parasitological parameters, **8** was more effective at ameliorating infection-induced hepatic fibrosis, significantly reducing mean granuloma diameter (66.7% vs. 30.8% for PZQ) and fibrotic areas (86.3% vs. 69.2% for PZQ) relative to the infected control group [160]. Furthermore, immunohistochemical and serological analysis showed that **8** inhibited hepatic stellate cells (HSCs) activation through the down-regulation of nuclear factor kappa-light-chain-enhancer of activated B cells (NF-κB), transforming growth factor (TGF)-β1, α-smooth muscle actin (α-SMA), and serum interleukin (IL)-13 fibrotic marker expression, together with the up-regulation of caspase-3 and p53 apoptotic marker expression and increased serum tumor necrosis factor (TNF)-α, contributing to the regression of *S. mansoni*-induced liver fibrosis [160].

Other studies showed that **8** inhibited TGF-β1-mediated collagen production induced by *S. japonicum* soluble egg antigen (SEA) in mouse peritoneal macrophages in vitro [162], and reduced the size of *S. japonicum* egg-induced granulomas in infected mice by inhibiting TGF-β1 secretion as well as the proliferation and activation of HSCs, thereby decreasing α-SMA and collagen I expression [163]. **8** also ameliorated liver granuloma and fibrosis in *S. japonicum*-infected mice through down-regulation of IL-13 signaling in both HSCs [164] and alternatively activated macrophages (AAMs) [165], reducing collagen I production from HSCs and inhibiting alternative activation of macrophages and arginase-1 activity by blocking phosphorylation of Janus-activated kinase 2 (JAK2) and signal transducer and activator of transcription 6 (STAT6) [164,165], and reducing IL-13 secretion [165,166]. Contrary to **8**-treated *S. mansoni*-infected mice, no significant changes in worm burden and egg load were observed in *S. japonicum*-infected mice treated with **8** [165,166]. Nevertheless, these findings underscore the potential of **8** as a prophylactic agent against *S. japonicum*-induced hepatic fibrosis.

Another study evaluated the inhibitory activity of **8** against *S. mansoni*-induced hepatic angiogenesis, in comparison with AM and PZQ, in a murine model of schistosomiasis [167]. Angiogenesis is an early event in the fibrotic cascade, contributing to periovular granuloma formation and periportal fibrosis in schistosomiasis. Mice harboring *S. mansoni* were orally administered **8** (50 mg/kg/day), AM (300 mg/kg/day), or PZQ (300 mg/kg twice daily) for one month, starting 6 weeks post-infection (before the onset of hepatosplenomegaly). Treatment resulted in a significant reduction in hepatic worm burden and in eggs per gram (OPG) levels in both feces and hepatic tissue compared with the non-treated control group, with drug efficacy increasing in the order AM < PZQ < **8** [167]. Likewise, **8** was more effective than PZQ and AM in reducing mean granuloma diameter and fibrotic area. This marked decrease in hepatic fibrosis was associated with lower α-SMA expression in pericytes relative to AM and PZQ treatments. Moreover, **8** significantly reduced the expression of pro-angiogenic vascular endothelial growth factor (VEGF), vascular proliferating cell nuclear antigen (PCNA; a marker of endothelial proliferation), and the proangiogenic marker CD34, while increasing anti-angiogenic tissue inhibitor of metalloproteinases 2 (TIMP-2) expression in myofibroblasts, fibroblasts and vascular endothelial cells, thereby strongly contributing to angiogenesis inhibition and fibrosis prevention [167].

In a different approach, the cercaricidal activity of hinokitiol (**9**; also known as β-thujaplicin), a tropolone monoterpenoid found in cupressaceous species such as western red cedar (*Thuja plicata*) and hinoki cypress (*Chamaecyparis obtusa*), was evaluated to assess its potential as a topical prophylactic agent against *S. mansoni* infection [168]. At concentrations above 25 μg/mL, **9** exhibited dose-dependent cercaricidal activity, characterized by reduced motility, impaired swimming, and a brown discoloration of the acetabular glands. The junction between the body and tail became fragile, although the tegument remained largely intact. These alterations effectively prevented cercariae from penetrating the host skin. However, neither single nor repeated topical applications of **9** to mouse skin (before or after cercarial exposure) reduced adult worm recovery, except when the compound was applied immediately prior to parasite challenge [168].

Transmission electron microscopy (TEM) revealed that **9** at 25 μg/mL induced progressive morphological alterations in *S. mansoni* cercariae, first detectable at 15 min post-exposure, including loss of the cercarial tail in approximately 50% of organisms [169]. This was followed by degeneration of the acetabular glands, loss of the external glycocalyx, and thinning of the tegument, which resulted in focal spine loss and outward protrusion. Edematous swelling of the muscle layer and deeper parenchyma subsequently developed, ultimately leading to membrane rupture after 120 min of exposure [169]. These structural changes likely account for the inability of **9**-treated cercariae to penetrate host skin [169].

Similarly, linalool (**10**), an acyclic monoterpene alcohol and the major constituent isolated from *Cinnamomum camphora* leaf extracts, exhibited potent larvicidal effects against *S. japonicum* cercariae (LC_50_ = 0.07 μg/mL at 6 h), inducing damage to the tegument and body–tail junction and leading to cercarial tail shedding 30 min after treatment with **10** at 0.1 μg/mL [170]. Furthermore, **10** at 0.1 and 1.0 μg/mL markedly reduced the number of schistosomula recovered from mouse skin after challenge infection. A significant reduction in total worm burden (60.75%), consistent with decreased hepatic (58.12%) and intestinal (64.90%) egg counts, was observed in infected mice 45 days after challenge with cercariae pretreated with 1.0 μg/mL of **10**. However, the number of tissue egg counts per female worm was equivalent between the treatment and control groups, indicating that **10** did not affect the fecundity of adult female worms [170]. In addition to its cercaricidal effects, **10** also exhibited molluscicidal activity against the intermediate host snail *Oncomelania hupensis* (LC_50_ = 0.25 μg/mL at 96 h) [170], which relates to vector control rather than direct parasite-targeted activity. The antischistosomal activity of plant-derived monoterpenoids is summarized in Table 2.

### 4.2. Sesquiterpenes

Among plant-derived sesquiterpenes, the sesquiterpene lactones (STLs) are best known for their anti-inflammatory, cytotoxic, antiparasitic, and antimicrobial properties [108,171,172,173,174,175,176,177,178]. Germacranolide-type and furanoheliangolide-type STLs isolated from several medicinal plants are particularly active due to their electrophilic α-methylene-γ-lactone moiety, which acts as a Michael acceptor and reacts with biological nucleophiles, such as thiol groups in cysteine residues [108,172,173,176,177,178].

**Figure 5:**
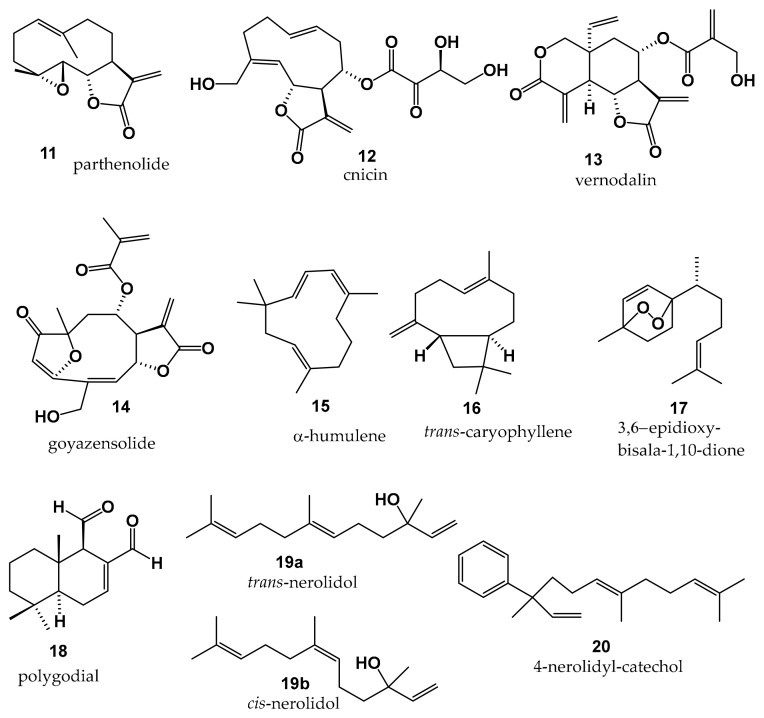
Antischistosomal plant-derived sesquiterpenes.

Plants of the genus *Tanacetum* (Asteraceae) have been used in traditional medicine to treat fever, migraine, neuralgia, rheumatism, and arthritis, and also as vermifuges and anthelmintics [174,175]. Extracts and EOs of *Tanacetum* species and their isolated metabolites have shown anti-inflammatory, antimicrobial, antiprotozoal, and anthelmintic activities [174,175,179,180,181,182,183,184,185]. Parthenolide (**11**; Figure 5), the main bioactive component of *Tanacetum parthenium* (feverfew), is a germacranolide STL with trypanocidal [181] and leishmanicidal [185] activities. Parthenolide (**11**) has also shown in vitro schistosomicidal activity (LC_50_ = 9.5 μM at 72 h), reducing the motor activity of adult *S. mansoni* and separating worm couples into individual males and females, thereby preventing oviposition [186]. The compound achieved 100% mortality after 48 h of incubation at 12.5 μM without affecting mammalian cells [186]. Confocal laser scanning microscopy revealed morphological alterations in the schistosome tegument, including sloughing and collapsed or disrupted tubercles, which correlated with parasite death [186].

Although the precise mechanism underlying the antischistosomal effects of **11** remains unclear, it has been reported that the α,β-unsaturated carbonyl structures of STLs, such as the α-methylene-γ-lactone present in **11**, contribute to their antiparasitic activity by reacting with nucleophiles via Michael-type addition, particularly the exposed sulfhydryl groups of cysteine residues found on the tegument surface and tubercles of schistosomes, leading to morphological alterations and inhibition of tegumental enzymes [176,178,179]. The antitrypanosomal and antileishmanial activities of **11** have been associated with loss of membrane integrity and mitochondrial dysfunction in *Trypanosoma cruzi* trypomastigotes [181], and *Leishmania amazonensis* amastigotes [185,187].

Cnicin (**12**), another germacranolide STL isolated from the leaf extract of blessed thistle (*Centaurea benedicta*), also from the Asteraceae family, has been shown to exhibit in vitro antiparasitic activity, notably antileishmanial [171,176] and [178] trypanocidal effects. When evaluated against *S. mansoni*, **12** at 25 and 50 μM caused 100% reduction in motor activity and 100% lethality of adult worms after 24 h of incubation. Similar effects were observed in adult female worms treated with 6.5 μM cnicin for 48 h, whereas male worms were unaffected [188]. Higher susceptibility of female schistosomes has also been observed with other antischistosomal STLs, including the artemisinin semi-synthetic derivatives AM, AS, DHA, and artemisitene [71,91,189,190]. On the other hand, **12** showed moderate cytotoxicity toward murine macrophages (CC_50_ = 21.83 μM) in vitro [188], consistent with its reported antiproliferative effects in human cancer cell lines [177], likely attributable to its reactive α-methylene-γ-lactone moiety [177].

In mice harboring adult *S. mansoni*, oral administration of **12** (100 mg/kg) produced no significant reduction in worm burden, whereas i.p. administration (10 mg/kg) decreased total worm load by 41.9% compared with the untreated control group [188]. Both treatments resulted in separation of adult worm pairs into individual males and females. However, neither route of administration significantly affected the number of OPG in feces. The low oral bioavailability of **12**, as a result of its poor aqueous solubility and extensive first-pass hepatic metabolism, may account for the greater efficacy observed with i.p. administration relative to oral dosing [188].

*Vernonia amygdalina* (Asteraceae), commonly known as bitter leaf plant due to the bitter taste of its leaves, is an African medicinal plant rich in cytotoxic furanoheliangolide STLs, like vernodalin (**13**), and steroid glucosides with antischistosomal activity, presumably used by wild chimpanzees for self-medication against parasite-related diseases [191,192,193]. In vitro, **13** was shown to inhibit adult *S. japonicum* motility and oviposition at approx. 20 μg/mL compared to 2 μg/mL for PZQ [192]. However, oral administration of **13** to *S. japonicum*-infected mice at 120 mg/kg was lethal, and lower doses (60 mg/kg) had no significant schistosomicidal activity [192].

Goyazensolide (**14**), another furanoheliangolide STL responsible for the schistosomicidal properties of *Eremanthus goyazensis* (Asteraceae), reduced egg production and motor activity of adult *S. mansoni* at concentrations above 0.8 μg/mL, reaching 90% mortality at 3.5 μg/mL after 24 h of incubation [194]. Female worms were more susceptible than males. Tegumental vesiculation was observed under the light microscope, and the damage to the worm surface was proportional to terpenoid concentration and time of exposure [194].

The bicyclic sesquiterpenes α-humulene (**15**; also known as α-caryophyllene) and *trans*-caryophyllene (**16**; also known as β-caryophyllene) showed in vitro schistosomicidal activity, with LC_50_ values of 149.98 μg/mL and 142.11 μg/mL, respectively, against *S. mansoni* adult worms [195]. No significant difference was observed in mortality between male and female parasites, with **15** and **16** causing 60% and 80% total worm mortality, respectively, after 72 h of incubation at 200 μg/mL. Both compounds reduced worm motor activity and decreased egg production at sublethal concentrations. Analysis of membrane integrity revealed lesions in the tegument and in the oral and acetabular suckers of both male and female worms exposed to the sesquiterpenes at 200 μg/mL, which may result in a reduced ability to adhere to blood vessels, thereby hindering nutrient ingestion [195].

Additionally, the effect of the bicyclic sesquiterpenes on the excretory system activity of *S. mansoni* was monitored using a resorufin probe [195]. In the control (untreated) group, both male and female worms showed complete tegumental absorption of the probe and accumulation in the excretory main tubules and lateral branches, indicative of normal excretory function. In resorufin-labeled female worms, exposure to the sesquiterpenes (200 μg/mL for 4 h) resulted in more accentuated fluorescence in the excretory main tubules and branches, suggesting that the treatment did not impair female excretory activity. On the contrary, sesquiterpene-treated male worms exhibited complete inhibition of excretory activity, characterized by blockage of resorufin flux in the excretory main tubules and branches [195].

Based on the potent activity of the hexane extract from branches of *Drimys brasiliensis* (Winteraceae) against *S. mansoni* (100% worm lethality at 200 μg/mL), bioactivity-guided fractionation yielded 3,6-epidioxy-bisabola-1,10-dione (**17**) as a diastereomeric mixture of endoperoxide bisabolane-type sesquiterpenes [196], and a drimane sesquiterpene, polygodial (**18**) [197], among other constituents. In vitro, **17** significantly reduced *S. mansoni* viability, with EC_50_ values of 4.1 μM against both male and female adult schistosomes, comparable to PZQ that showed EC_50_ values of 1.1 and 1.3 μM, respectively [196]. Separated analysis of adult worm pairs as male and female parasites is useful since biological and physiological differences between sexes are known to influence drug susceptibility. Similarly, **18** equally inhibited male and female *S. mansoni* worms, with EC_50_ values of 9.6 ± 0.5 μM and 9.3 ± 1.7 μM, respectively [197]. Cytotoxicity assays against mammalian cells revealed no significant toxicity for either compound (CC_50_ > 200 μM) [196,197]. Additionally, in silico analysis using the SwissADME platform predicted good drug-likeness properties for both sesquiterpenes, comparable to those of the reference drug PZQ [196,197].

In *S. mansoni*-infected mice, treatment with a single oral dose (400 mg/kg) of either **17** or **18** reduced total worm burden by 61.2% and 44.0%, respectively [196,197]. A decrease in egg production of 69.5% in intestinal tissues and 71.8% in fecal samples was also observed for **18**-treated mice [197]. A remarkable egg burden reduction of 98.2% in intestinal tissues and 99.2% in feces was achieved in **17**-treated animals, surpassing the efficacy of PZQ, which reduced egg counts by 84.1% and 89.8% in intestines and feces, respectively [196]. This finding is noteworthy because egg production is the primary driver of schistosomiasis-associated morbidity and transmission [197].

Nerolidol (**19**), an acyclic sesquiterpene alcohol found in the EOs of several plants [198], displays antioxidant [199,200,201,202,203], antimicrobial [200,204,205,206,207], and broad antiparasitic activities, including antimalarial biofilm [138,200,208,209,210,211,212,213], antileishmanial [214,215,216,217], antitrypanosomal [218,219,220], and antibabesial [221] effects.

Nerolidol (**19**), used as a 1:1 mixture of *trans*-nerolidol (**19a**) and *cis*-nerolidol (**19b**), exhibited in vitro activity against 49-day-old adult *S. mansoni* (BH strain). At 15.6 μM, the lowest concentration tested, it promoted the separation of all paired adult worms into individual males and females [222]. At 31.2 μM and 62.5 μM, **19** reduced the motor activity of male and female worms, respectively. At 62.5 μM, **19** was lethal to 100% of male worms after 48 h of incubation, whereas no mortality was observed in females, indicating greater susceptibility of males [222]. At 125 μM, **19** significantly reduced motor activity and caused 100% mortality of male worms at 24 h and female worms at 48 h. Confocal laser scanning microscopy revealed marked tegumental alterations, including disintegrated tubercles, sloughing, and surface erosion, with a strong correlation between tegumental damage and loss of viability [222].

On the contrary, *trans*-nerolidol (**19a**), the major constituent of the EO from *Baccharis dracunculifolia* leaves, was inactive against 56-day-old adult *S. mansoni* (LE strain) at 10–100 μM, despite the EO itself showing potent in vitro schistosomicidal activity [223]. The EO reduced motor activity and induced complete parasite at 10 μg/mL after 24 h and caused tegumental damage at 100 μg/mL, whereas pure **19a** produced no morphological alterations [223]. These discrepancies may reflect strain-dependent differences in nerolidol susceptibility and raise the possibility that *cis*-nerolidol (**19b**) is the more active isomer [222].

Further in vivo studies in a mouse model of schistosomiasis infected with either adult (patent) or juvenile (prepatent) stages of *S. mansoni* showed that a single oral dose of **19** (100, 200 or 400 mg/kg) administered to mice harboring adult worms led to significant reductions in worm burden and egg production [224]. At the highest dose (400 mg/kg), reductions of 70.1% in worm burden, 84.6% in the immature egg count, and 75.2% in fecal egg load were achieved. The decreased egg production may reflect **19**-induced reductions in worm burden and/or inhibition of oviposition by mature worm pairs. SEM analysis of adult worms recovered from **19**-treated mice revealed tegumental alterations in both male and female worms, including focal damage of the dorsal surface. In males, tubercle swelling and shortening or loss of spines were also observed. These findings suggest that **19**-mediated parasite killing is associated with tegumental damage [224].

However, **19** showed low efficacy in the treatment of juvenile *S. mansoni*, even at 400 mg/kg, in marked contrast to its activity against adult worms [224]. In vitro evaluation of **19** against different developmental stages of *S. mansoni* revealed LC_50_ values of 117.08, 124.62 and 84.99 μM for newly transformed schistosomula (NTS), juvenile (21-day-old), and adult (49-day-old) worms, respectively, confirming that **19** is more potent against the adult stage, consistent with the in vivo findings [224].

Pharmacokinetic studies in mice showed that a single oral dose of 1000 mg/kg nerolidol (**19a**/**19b** 3:1 *w*/*w*) produced a plasma concentration of 0.27 ± 0.07 μg/mL within 30 min of administration, which remained stable for 3 h and reached a maximum of 0.35 ± 0.05 μg/mL at 6 h. By 12 h after dosing, plasma levels of **19** had decreased to nearly zero [225]. Another pharmacokinetic study in rats reported a maximum plasma concentration of 8.30 ± 1.07 μg/mL at 20 min after a single intraperitoneal injection of 25 mg/kg nerolidol (**19a**/**19b** 3:2), which declined to nearly zero within 2 h of administration [226]. The lower peak plasma concentration of **19** after oral administration may result from first-pass hepatic metabolism, which reduces oral bioavailability compared with intraperitoneal injection [198].

Acute toxicity studies reported LD_50_ values for **19** greater than 5000 mg/kg following oral administration in rats and mice and dermal administration in rabbits, indicating that the compound is practically non-toxic by both the oral and transdermal routes [121]. In human dermatological studies, topical application of 4% nerolidol (**19**) under occlusion for 48 h did not induce skin irritation, and no positive reactions were observed in maximization or repeated-insult patch tests, indicating an absence of skin-sensitizing potential [121].

Based on favorable parasitological parameters, pharmacokinetic profile, and toxicological data, **19** has therapeutic potential as an antischistosomal agent with additional antiprotozoal activities. The importance of the nerolidol scaffold for antischistosomal activity is evident in the remarkable in vitro schistosomicidal potency of 4-nerolidylcatechol (**20**), which displays an EC_50_ of 0.91 μg/mL (2.9 μM) against adult *S. mansoni* and high selectivity (SI > 68.9) [227]. This meroterpenoid, isolated from the roots of *Pothomorphe umbellata* (Piperaceae), a species used in African and South American traditional medicine for treating malaria and helminthiasis, was also effective in vivo in mouse models of both prepatent and patent *S. mansoni* infections. A single oral dose (400 mg/kg) reduced worm burden and egg production, and attenuated hepatomegaly and splenomegaly [227]. Collectively, these findings highlight the nerolidol scaffold as a promising lead for the development of potent and selective antischistosomal agents. The antischistosomal activity of plant-derived sesquiterpenoids is summarized in Table 3.

### 4.3. Diterpenes

**Figure 6:**
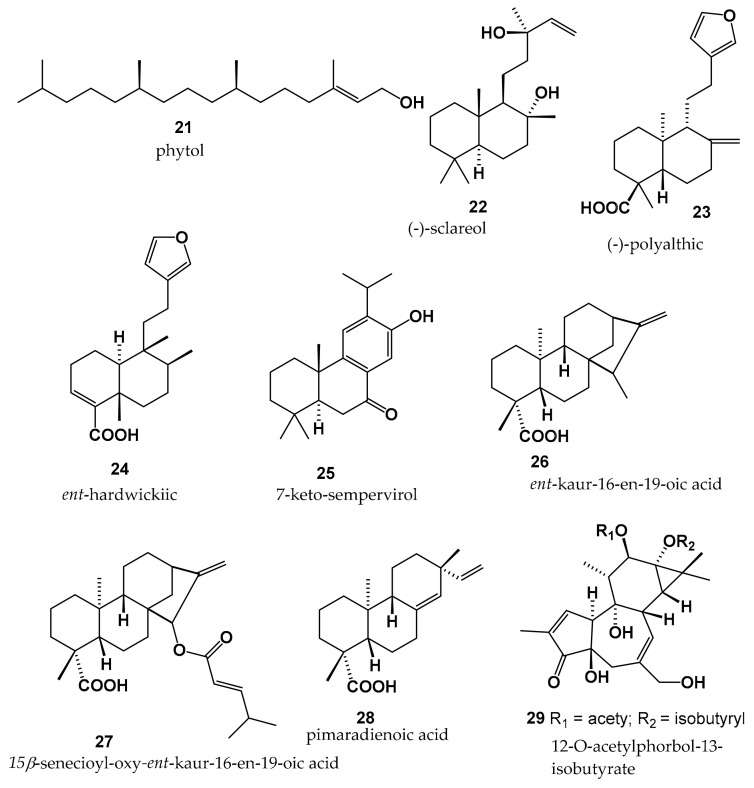
Antischistosomal plant-derived diterpenes.

Phytol (**21**; Figure 6), an acyclic diterpene alcohol best known as the side chain of chlorophyll, represents one of the simplest members of the diterpene family. It can be readily obtained by hydrolysis of chlorophyll extracted from plant biomass and serves as a key biosynthetic precursor to several biologically active metabolites, including tocopherols and phytyl diphosphate-derived compounds [228,229]. Phytol (**21**) was found to reduce the motor activity of *Schistosoma mansoni* female worms in vitro at 50 μg/mL, causing their death within 24 h, while a concentration of 100 μg/mL was required to kill male worms in the same period [228]. According to confocal laser scanning microscopy, the diterpene induced extensive tegumental alterations in a concentration-dependent manner, with female worms being more susceptible than males. PZQ (1 μg/mL) used as a positive control caused severe muscle contractions and produced tegumental changes in 100% of the worms after 24 h, in marked contrast to **21**, which induced paralysis but not muscle contraction [228]. At sublethal doses (25 μg/mL), **21** impaired the reproductive fitness of *S. mansoni*, causing a 75% reduction in egg production compared with untreated controls. In mice harboring adult *S. mansoni*, oral administration of a single dose of **21** (40 mg/kg) at 56 days post-infection resulted in a 51.2% reduction in total worm burden (70.3% in female worms) and a 76.6% decrease in fecal egg counts [228]. Confocal microscopy of adult worms recovered from treated mice revealed tegumental damage, particularly in females, consistent with the earlier in vitro findings [228].

Phytol (**21**) exhibited in vitro schistosomicidal activity against juvenile and adult *S. haematobium* in a dose- and time-dependent manner. At low concentrations (<100 μg/mL), the compound significantly reduced worm motility, achieving 100% lethality after 48 h of incubation at 150 μg/mL. Male worms, at both juvenile and adult stages, were more susceptible than females [230], contrasting with previous reports indicating greater sensitivity of *S. mansoni* adult females to **21** [228]. SEM revealed ultrastructural alterations in immature and mature stages of *S. haematobium*, including tegumental peeling, tubercle disintegration, unfolding and widening of the gynecophoral canal, and damage to oral and ventral suckers [230]. Although the precise mechanism underlying the schistosomicidal activity of **21** remains unclear, the extent of tegumental disruption strongly correlated with reduced worm viability [230].

Sclareol (**22**), an antimicrobial labdane diterpenoid from the aromatic herb *Salvia sclarea*, was shown to affect motility and the phenotype of *S. mansoni* schistosomula with IC_50_ values of 12.3 μM and 14.2 μM, respectively [231]. The compound also inhibited motor activity of juvenile (IC_50_ = 5.5 μM) and adult (IC_50_ = 19.3 μM) worms, while displaying low toxicity toward human cells (CC_50_ = 74.1 μM in the HepG2 cell line). Furthermore, **22** reduced egg production by *S. mansoni* adult worm couples compared with the untreated control group, even at the lowest concentration tested (3.13 μM) [231]. Further structure–activity relationship (SAR) analysis of semi-synthetic derivatives of **22** revealed that the free allylic alcohol was essential for anthelmintic activity [231]. The most potent compound was a Heck-coupled derivative bearing a 4-substituted phenyl ring, which exhibited improved antischistosomula activity, likely due to increased lipophilicity and enhanced membrane permeability [231]. SEM and metabolomics analyses suggested that this sclareol analogue acts primarily by perturbing surface membranes and disrupting membrane lipid homeostasis through interference with arachidonic acid metabolism [231], a mechanism clearly distinct from the metabolic profile elicited by PZQ, which involved alterations in carbohydrate metabolism [231] consistent with its previously reported effects on serotonin signaling [232,233] and the glycolytic pathway [234].

(−)-Polyalthic acid (**23**), another labdane-type diterpene isolated from the oleoresin of Brazilian *Copaifera duckei*, exhibited in vitro schistosomicidal activity with an LC_50_ value of 107.9 μM (33.4 μg/mL) after 72 h of incubation with *S. mansoni* adult worms [235]. Moreover, the compound significantly inhibited egg development in a dose-dependent manner and completely suppressed egg production within 72 h at concentrations above 100 μM [235].

Among the labdane-, clerodane-, and kaurane-type diterpenes isolated from *Copaifera* oleoresins and their derivatives obtained by microbial transformation, *ent*-hardwickiic acid (**24**) showed promising in vitro schistosomicidal activity [236]. This natural *ent*-labdane diterpenoid displayed an IC_50_ value of 25.7 μM after 72 h of incubation and achieved 100% lethality of adult *S. mansoni* at 200 μM after 24 h. Extensive tegumental damage in both male and female worms was observed under an inverted microscope [236].

Furthermore, **24** promoted the separation of 100% of the coupled adult worms into individual males and females after 24 h of incubation at concentrations ranging from 25.0 to 100.0 μM, completely inhibiting egg development [236]. At lower sublethal concentrations (6.25 and 12.5 μM), the compound inhibited egg production by more than 80% after 72 h of incubation and induced the premature release of non-viable oocytes into the culture medium. These findings could not be attributed to worm pair separation, which occurred only at concentrations of 25 μM and above, suggesting that **24** can interfere with the reproductive system of female adult *S. mansoni* [236], similarly to other diterpenes such as phytol (**21**) [228] and *ent*-polyalthic acid (**23**) [235].

Plants from the *Lycium* genus (Solanaceae family) have long been used as medicinal and edible species in East Asia [237]. Among them, *Lycium barbarum* and *L. chinense* are widely employed in Traditional Chinese Medicine (TCM) due to their anti-ageing, antioxidant, vision-enhancing, hepatoprotective, anti-inflammatory, and immunomodulatory effects [237,238]. Their fruits, commonly known as goji or wolfberry, are considered functional foods owing to their nutritional value and antioxidant properties [238].

The clerodane-type diterpenoid 7-keto-sempervirol (**25**), isolated from the root of *Lycium chinense*, displayed dual in vitro anthelminthic activity against the trematodes *S. mansoni* and *F. hepatica* [239]. The compound was effective against *S. mansoni* schistosomula (LD_50_ = 19.1 μM) and *F. hepatica* newly excysted juveniles (LD_50_ = 17.7 μM), and also affected adult *S. mansoni*, reducing worm motility and inducing tegumental damage [239]. SEM imaging revealed surface holes, tubercle swelling, and spine shortening or loss in adult male *S. mansoni* treated with **25**, phenotypes that were also observed in treated adult *F. hepatica* [239]. The compound further inhibited oviposition, eggshell formation, and normal egg maturation. Laser scanning confocal microscopy of adult females exposed to **25** showed non-oval, irregularly shaped in utero eggs lacking fully formed eggshells and the characteristic lateral spines [239].

This diterpene represents a promising scaffold for simultaneously targeting schistosomiasis and fascioliasis. SAR analysis of synthetic 7-keto-sempervirol analogues designed to improve the diterpenoid potency and selectivity revealed several trends. Non-cyclized derivatives lacking the C9-C10 bond showed reduced anti-schistosomula activity. In contrast, enhanced activity was observed upon removal of the hydroxyl group and upon replacing the isopropyl moiety with a methoxy group, or simply by removing both the isopropyl and 7-keto groups from the natural diterpenoid [231].

The *ent*-kaurane diterpenes isolated from aerial parts of *Baccharis lateralis* (Asteraceae), *ent*-kaur-16-en-19-oic acid (**26**) and 15β-senecioyl-oxy-*ent*-kaur-16-en-19-oic acid (**27**) exhibited in vitro activity against *S. mansoni*, with LC_50_ values of 26.1 and 11.6 μM, respectively, after 72 h of incubation [240]. The diterpenes induced parasite death in a time- and concentration-dependent manner. At 25 μM, compound **27** achieved 100% mortality after 24 h of incubation while **26** at the same concentration required 48 h to produce the same effect. Compound **27** exhibited lower cytotoxicity to human cell lines than **26**, indicating greater selectivity for the parasite. The presence of the senecioloxy group in **27** may have contributed to the differences in the in vitro antischistosomal activity and cytotoxicity of the compounds [240].

A pronounced reduction in oviposition was also observed after 72 h treatment of mated adult worms with **27** at concentrations from 25 to 6.25 μM whereas **26** at the highest concentration tested (25 μM) only produced a moderate effect in egg load reduction [240]. The decline in egg production was closely linked to loss of male/female pairing. The effects of the diterpenes on the morphology of reproductive organs of female and male adult worms were evaluated by microscopy analysis after indigo carmine staining. Signs of dilation in both testes and ovaries were observed in worm couples treated with **27** while **26** did not cause significant alterations in the male and female reproductive systems [240]. Due to its higher in vitro antischistosomal efficacy and better selectivity profile (SI > 10), **27** was selected for further in vivo studies in a chronic murine model of schistosomiasis.

In mice harboring patent *S. mansoni* infections, a single oral dose of **27** (400 mg/kg) significantly reduced total worm burden by 61.9% and decreased the number of immature eggs in the intestinal wall and fecal samples by 69.2% and 71.6%, respectively [240]. The decline in egg numbers may reflect either a treatment-induced reduction in adult schistosome burden or a suppression of female oviposition. Additionally, organ pathology associated with *S. mansoni* infection, assessed by liver and spleen weight measurements, indicated that **27** ameliorated hepatosplenomegaly relative to untreated infected mice, presumably due to reductions in worm and egg burdens [240].

Pimaradienoic acid (**28**), a pimarane-type diterpenoid and the major constituent isolated from the root extract of *Viguiera arenaria*, exhibited antischistosomal activity, significantly reducing the viability of adult *S. mansoni* in vitro at concentrations between 25 and 100 μM after 120 h of incubation and without cytotoxicity toward human fibroblasts (CC_50_ > 500 μM) [241]. The compound induced morphological alterations of the worm’s tegument at 100 μM after 24 h incubation, separated coupled adult worms in a concentration- and time-dependent manner, and significantly reduced egg production at 50 μM, presumably due to worm couple separation. Inhibition of egg development was also observed at all tested concentrations (10–100 μM). Further in vivo preliminary studies showed that treatment of infected mice with **28** (100 mg/kg, i.p.) reduced total parasite burden by 40% in infected mice [241].

The seeds of *Croton tiglium* (Euphorbiaceae, subfamily Crotonoideae), known as *Crotonis Fructus*, have been used in traditional medicine for the treatment of cancer, gastrointestinal diseases, peptic ulcer, and helminth infections, among other ailments [242]. Several tigliane-type diterpenes with schistosomicidal activity have been isolated from the leaves of *C. tiglium*, whose extracts possess anti-schistosomiasis liver fibrosis effects [19]. Among those, the phorbol ester 12-*O*-acetylphorbol-13-isobutyrate (**29**) showed 100% mortality of schistosomula after 72 h at 8.5 μg/mL (the lowest concentration assayed) and additional in vitro anti-fibrosis effects [19]. The diterpenoid significantly downregulated the mRNA levels and protein expression of Col-I, Col-III, α-SMA, and TGF-β1 on TGF-β1-induced liver fibrosis in LX-2 cells compared with the model group. The phorbol ester exerted its anti-fibrotic effects through regulating the TGF-β1/Smad signaling pathway by reducing the mRNA and protein expressions of TGF-βRI, TGF-βRII, Smad2, and Smad3 [19]. The antischistosomal activity of plant-derived diterpenoids is summarized in Table 4.

### 4.4. Triterpenes

**Figure 7:**
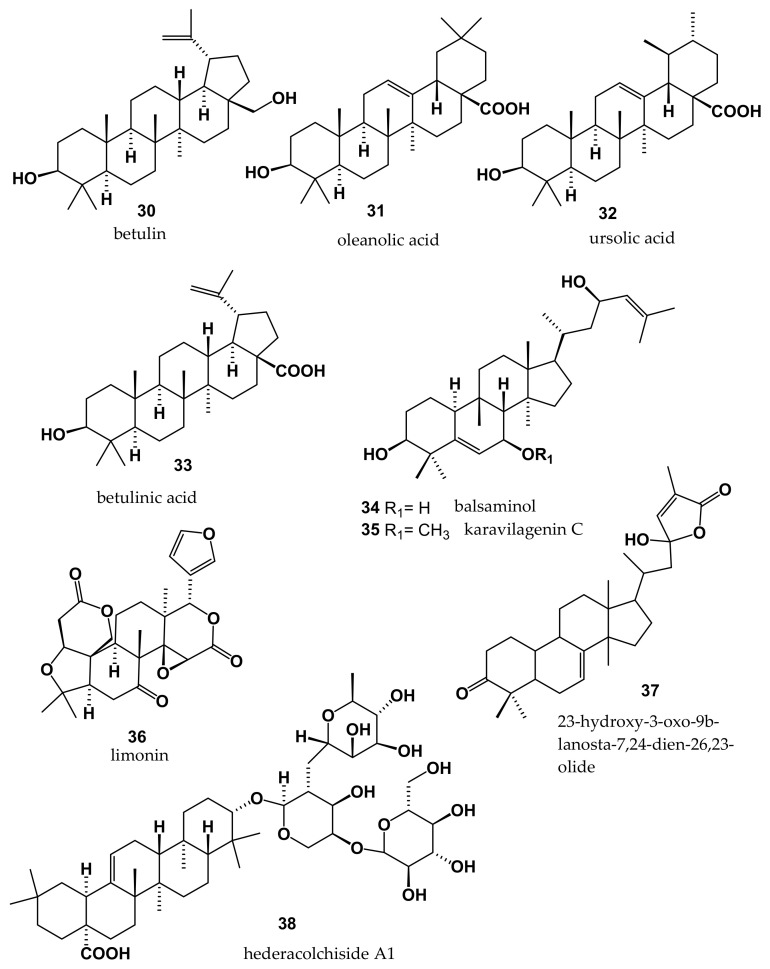
Antischistosomal triterpenes.

Preliminary in vitro studies on the antischistosomal activity of some Brazilian Cerrado species and their isolated compounds identified several bioactive triterpenes and flavonoids [243]. Betulin (**30**; Figure 7) from *Schefflera vinosa* (Araliaceae), as well as oleanolic acid (**31**) and ursolic acid (**32**) from *Miconia langsdorffii* (Melastomataceae), reduced the motor activity of adult *S. mansoni* but did not induce tegumetal damage at 50, 100, or 200 μM, even after 5 days of exposure. However, **31** and **32**, previously identified as the metabolites responsible for the leishmanicidal activity of *M. langsdorffii* [244], did not produce lethal effects on *S. mansoni*. Only **30** caused parasite mortality, inducing 25% death at 200 μM after 24 h and 50% after 120 h. These findings suggest that the lupane skeleton, with its characteristic five-membered E-ring and isopropenyl group at C-19 and/or the primary hydroxyl group of **30**, may confer greater antischistosomal activity than the six-membered E-ring and/or the carboxyl groups present in **31** and **32** [243].

The relevance of the lupane skeleton for antischistosomal activity (rather than the additional primary hydroxyl group present in **30**) is supported by the observation that betulinic acid (**33**) emerged as the main active constituent among the compounds isolated from schistosomicidal *Eremanthus erythropappus* extracts [172]. Betulinic acid (**33**) exhibited in vitro antischistosomal activity against adult *S. mansoni* (EC_50_ = 36.8 μM) with no detectable cytotoxicity toward mammalian cells (CC_50_ > 500 μM) and good selectivity (SI > 15.5) [172]. In vivo, a single oral dose of **33** (400 mg/kg) administered to *S. mansoni*-infected mice reduced total worm burden by 41.9% [172].

*Momordica balsamina* (Cucurbitaceae), commonly known as balsam apple, balsam pear, or African cucumber, has both nutritional and medicinal properties and is used in African traditional medicine for the treatment of malaria, diabetes, and other ailments [245,246]. Both in vitro and in vivo studies have demonstrated antimalarial activity for *M. balsamina* extracts [245] and for their major constituents, the cucurbitane-type triterpenoids [247,248,249]. Among these, balsaminol F (**34**) and karavilagenin C (**35**) exhibit dual antimalarial [250,251] and antischistosomal [252] activities. Both compounds were similarly effective against blood-stage schizonts of chloroquine-sensitive (3D7) and chloroquine-resistant (Dd2) strains of *P. falciparum*, with IC_50_ values of 18.0 and 20.0 μM for **34** [250] and 10.4 and 11.2 μM for **35** [251], respectively. Both **34** and **35** displayed in vitro schistosomicidal activity against adult *S. mansoni*, with LC_50_ values of 14.7 and 28.9 μM, respectively, after 24 h of incubation [252]. The triterpenoids caused 100% lethality of adult worms after 24 h of incubation with **34** at 50 μM or **35** at 100 μM. At 10–50 μM, the compounds significantly reduced worm motility, decreased egg production, and inhibited egg development. Additionally, **34** at 25 μM and **35** at 50 μM were able to separate all paired adult worms after 24 h of incubation [252]. The higher antischistosomal activity of **34** was attributed to the free hydroxyl group at C-7, which is methoxylated in **35**. The importance of free hydroxyl groups in the triterpenoid skeleton for schistosomicidal activity was further supported by the lack of activity of the glycosylated derivatives of **34** and the acylated derivatives of **35** (C-3 and/or C-23 esters) [252]. On the contrary, free hydroxyl groups were associated with lower antimalarial activity, and acylation of **34** and **35**, such as in triacetylbalsaminol F [251] and karavoate B [251], resulted in enhanced antiplasmodial activity, particularly against the chloroquine-resistant strain [250,251]. These findings indicate that rational modification of cucurbitane triterpenoids can be used to modulate antiparasitic activity, increase potency, and improve selectivity. The evidence that some cucurbitane-type triterpenoids exhibit both antimalarial and antischistosomal activities suggests that they may target the heme detoxification pathway, which is shared by both parasites [246]. Similarly, interference with hemozoin formation underlies both the antiplasmodial and schistosomicidal effects of the antimalarial quinoline methanols quinine and quinidine [253].

Limonoids are tetranortriterpenoids mainly found in the Rutaceae and Meliacea plant families. These highly oxygenated triterpenoids are biosynthesized through the acetate–mevalonate (MVA) pathway and occur in significant amounts as aglycones and glycosides in seeds and fruit tissues [254]. Several limonoids have demonstrated antiprotozoal and anthelmintic activities, both in vitro and in vivo [255,256,257,258,259].

Limonin (**36**) is a bitter limonoid aglycone found in a wide range of citrus fruits, including bitter orange (*Citrus aurantium*), grapefruit (*Citrus paradisi*), lime (*Citrus aurantiifolia*), lemon (*Citrus limon*), sweet orange (*Citrus sinensis*), and pomelo (*Citrus maxima*), being typically present at higher concentrations in seeds [254]. The therapeutic efficacy of **36** was evaluated in experimentally infected mice harboring juvenile or adult *S. mansoni* [260]. A single oral dose of 50 or 100 mg/kg administered 21 days post-infection significantly reduced total worm burden by 70.0% and 83.3%, respectively, whereas the same dose given at 56 days post-infection produced reductions of 41.1% and 60.3%. Juvenile female worms were more susceptible than juvenile males, while in adult stages the opposite pattern was observed, with males showing greater susceptibility than females. The stage-dependent differences in susceptibility were attributed, in part, to the stronger antioxidant system of adult worms compared with juveniles. SEM analysis of male schistosomes revealed swollen or collapsed tubercles with complete loss of spines in juvenile worms, whereas adult worms exhibited disrupted tubercles with fewer and shorter spines, focal ulcerations, and tegumental peeling [260]. Additionally, marked reductions in hepatic and intestinal tissue egg loads were observed for both **36**-treated adult groups, accompanied by alterations in the oogram pattern characterized by increased proportions of dead eggs and a pronounced decrease in immature ova, which may reflect the reduction in worm burden [260]. Treatment with **36** also ameliorated infection-induced hepatic fibrosis, decreasing both the size and number of granulomas, an effect likely related to the terpenoid impact on schistosome viability and fecundity, which in turn reduces egg output and tissue egg deposition [260]. The antioxidant properties of **36** further contribute to its hepatoprotective effects, suppressing inflammation and oxidative stress [261]. Limonin (**36**) has been shown to activate the Nrf2 antioxidative pathway in the liver, reversing glutathione depletion and ROS accumulation, and to inhibit the NF-κB-mediated inflammatory signaling, thereby reducing macrophage infiltration and limiting the production of pro-inflammatory cytokines TNF-α, IL-6, and IL-1β [261,262]. Moreover, the compound attenuated TGF-β-induced hepatocyte epithelial–mesenchymal transition (EMT) and HSC activation in vitro, and mitigated CCl_4_-induced liver fibrosis in mice [263].

Among the ten triterpenoids isolated from the mature bark of *Abies* species (fir trees) indigenous to Wales (UK), the tetracyclic lanostane-type triterpenoid **37** (23-hydroxy-3-oxo-9β-lanosta-7,24-dien-26,23-olide) exhibited potent in vitro anthelmintic activity against both liver flukes (*Fasciola hepatica*) and blood flukes (*S. mansoni*), with EC_50_ values ranging from 0.7 to 15.6 μM [264]. Preliminary SAR analysis indicated that the tetracyclic steroid-like core and the flexible lactone side chain were associated with the observed antiparasitic effects. The triterpenoid was active against *S. mansoni* schistosomula (EC_50_ = 1.9 μM), juveniles (EC_50_ = 3.4 μM), and adult flukes (EC_50_ of 7.4 μM for males and 10.3 μM for females) based on motility after 72 h of incubation, and displayed anthelmintic selectivity relative to representative mammalian cell lines, with SI values in the range 3.1–16.6 [264]. The compound also decreased egg production at sublethal concentrations, which correlated with a loss of male–female pairing, and it inhibited somatic stem cell (neoblast) proliferation, with males being more susceptible than females. These findings suggest that **37** induced a stress response in adult schistosomes that significantly affected pairing, oviposition, and neoblast proliferation [264]. SEM analysis revealed tegumental surface disruption and membrane blebbing in both *S. mansoni* males and females exposed to a sublethal concentration of **37** (10 μM) for 72 h [264]. The antischistosomal activity of plant-derived triterpenoids is summarized in Table 5.

### 4.5. Triterpenoid Saponins

The rhizome of *Pulsatilla chinensis* (Ranunculaceae), a perennial herb used in traditional Chinese medicine, is widely employed as an adjunctive treatment for enteritis, bacterial dysentery, intestinal amebiasis, giardiasis, malaria (in combination with other herbs), vaginal trichomoniasis (as external wash), and certain malignant tumors, owing to its antimicrobial, anti-inflammatory, and immunomodulatory properties [189,265,266,267,268]. Triterpenoid saponins are the major bioactive constituents of *P. chinensis* [267,269,270].

**Figure 8:**
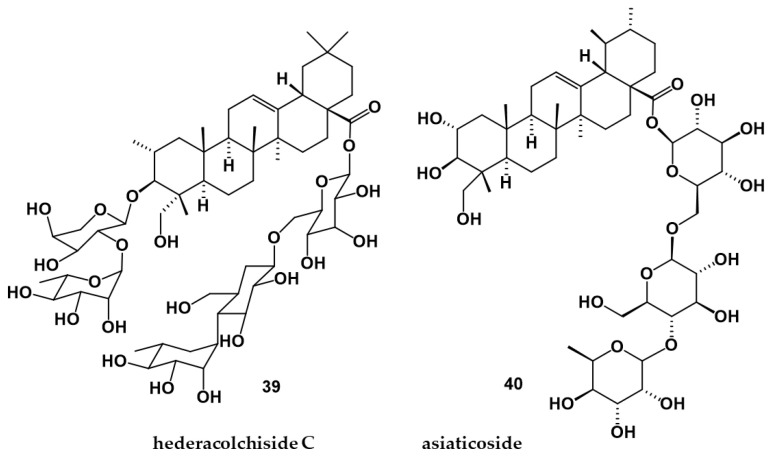
Antischistosomal triterpenoid saponins.

Hederacolchiside A1 (**38**; Figure 8), an oleanane-type triterpenoid saponin derived from the aglycone hederagenin isolated from *P. chinensis* exhibited in vivo antischistosomal activity against both juvenile and adult *S. japonicum* and *S. mansoni* [271]. Intraperitoneal administration of **38** (8 mg/kg) produced significant and comparable reductions in total and female worm burdens in mice infected with either juvenile or adult *S. japonicum* or *S. mansoni*. The saponin was particularly active against 1-day-old and 7-day-old schistosomes, being more potent than the reference drugs PZQ and AS at these stages. In mice harboring 1-day-old *S. japonicum* or *S. mansoni* (i.e., NTS), **38** (8 mg/kg, i.p.) achieved total worm burden reductions of 97.2% and 88.6%, respectively, compared with 62.4% and 86.2% for PZQ (300 mg/kg), and 59.3% and 64.4% for artesunate (300 mg/kg) [271].

Additionally, **38** (8 mg/kg, i.p.) reduced egg load in both juvenile and adult *S. japonicum*-infected mice, achieving high egg-reduction rates against 1-day-old (99.3%) and 7-day-old (98.7%) worms, and ameliorated liver damage [271]. **38**-treated mice showed reduced hepatic granulomatous inflammation, with decreased liver (and spleen) indexes (organ weight/body weight) and significant reductions in granuloma size and number compared with untreated mice. These effects were more pronounced in mice infected with *S. japonicum* NTS, which also exhibited decreased serum levels of the cytokines TNF-α, IL-4, and IL-17A, all involved in *Schistosoma*-induced granuloma formation [271]. **38** further inhibited the expression of the fibrotic proteins TGF-β1, TIMP-1, and collagen I in the mouse liver [271]. In vitro assays with *S. japonicum* NTS showed that **38** at 8 μg/mL (8.93 μM) achieved a 100% death rate after 48 h incubation, compared with 71.0% for 30 μg/mL PZQ (96.0 μM) and 94.6% for 30 μg/mL AS (78.0 μM) [271]. SEM analysis revealed that **38** at 8 μg/mL caused extensive tegumental disruption, including sloughing and erosion, contributing to its inhibitory effect against *S. japonicum* NTS [271].

Pharmacokinetic studies in rats indicate that **38** exhibits a relatively long elimination half-life (*T*_1/2_ ~ 8.8 h) after intravenous administration (0.5 μmol/kg), a feature that was also observed following oral dosing (20 μmol/kg and 200 μmol/kg), consistent with its low clearance and small volume of distribution [272]. Despite its slow systemic elimination, **38** displays very low oral bioavailability, likely due to poor gastrointestinal absorption associated with its large molecular weight and high polarity, which limit membrane permeability, and/or extensive first-pass metabolism. Further studies using the Caco-2 monolayer model showed that absorptive permeability (3.09 ± 0.33 × 10^−6^ cm/s) was significantly higher than efflux permeability (0.61 ± 0.20 × 10^−6^ cm/s), indicating that low oral bioavailability is driven primarily by weak transcellular permeation rather than active efflux [272]. Uptake of **38** was significantly reduced by phloridzin, a functional inhibitor of sodium-glucose linked transporter 1 (SGLT1) in Caco-2 cells, indicating that apical SGLT1 contributes to its limited active absorption across the intestinal epithelium [272].

Hederacolchiside C (**39**), another hederagenin-type triterpenoid saponin isolated from the roots of *P. chinensis*, exhibited in vitro schistosomicidal activity against adult *S. japonicum*, killing all worms after 72 h of incubation at 60 μg/mL [273]. SEM revealed extensive tegumental damage characterized by swelling, erosion, and peeling, which was more pronounced in female worms, where tegumental peeling exposed the underlying tissues [273]. Further in vivo studies demonstrated that intravenous (i.v.) **39** exerted dose-dependent antischistosomal, anti-inflammatory, and immunomodulatory effects in *S. japonicum*-infected mice [273]. The i.v. route was chosen due to the low bioavailability of **39** upon oral administration (less than 5%) [273]. Administration of multiple i.v. doses of **39** (100, 200, and 400 mg/kg, twice daily) for 5 consecutive days to mice harboring juvenile (14-day-old) or adult (35-day-old) *S. japonicum* reduced total worm burden by 28.9–44.1% and 27.9–48.1%, respectively, depending on dosage [273]. Females were more susceptible than males, with reduction rates of 30.7–49.2% and 38.7–62.5% in the juvenile and adult populations, respectively. The reduction in female worm burden was accompanied by hepatic egg load decreases of 39.9–58.6% in juvenile and 25.5–63.5% in adult *S. japonicum*-infected mice following **39** treatment [273]. In vivo cytotoxicity studies in mice treated with **39** (5.0 g/kg, i.v.) showed no changes in body weight compared with the untreated control group, and no mortality was observed during the 14-day assessment period, indicating that the compound was safe and well tolerated [273]. Histopathological examination of the livers of untreated *S. japonicum*-infected mice revealed large fibrocellular granulomas, which were reduced in both number and size in **39**-treated animals [273]. Comparison of immune responses before and after treatment with **39** showed that the compound decreased serum IgG levels and reduced the expression of the cytokines TNF-α, IL-4, and IL-17A, key mediators of the granulomatous inflammatory response in infected mice. In contrast, administration of **39** to uninfected mice produced no significant effects, suggesting that the reduction in cytokine expression observed in *S. japonicum*-infected mice following saponin treatment may result from suppression of the immunologic response that drives granuloma formation [273].

Nucleoside triphosphate diphosphohydrolases (NTPDases), also known as adenosine triphosphate diphosphohydrolases (ATPDases) or apyrases, hydrolyze several nucleoside tri- and diphosphates to the corresponding nucleoside monophosphates in the presence of divalent cations, such as calcium and magnesium [274]. In mammals, NTPDases function as key ecto-nucleotidases involved in the control of extracellular ATP/ADP signaling and have been implicated in immune regulation, thrombosis, and inflammation [274]. NTPDases have recently been identified on the surface of several parasites, including *Schistosoma* spp., where they participate in purine salvage, modulation of host extracellular ATP/ADP levels, immune evasion, and nutrient acquisition [274]. These tegumental ecto-enzymes, exposed on the parasite’s outer surface, contribute to parasite growth, infectivity, and virulence [274]. Consequently, *Schistosoma* spp. NTPDases represent attractive targets for the development of new antischistosomal agents.

Screening of NTPDase inhibitors from the hydroalcoholic extract of *Centella erecta* (Apiaceae) using potato apyrase identified the pentacyclic ursane-type triterpenoids madecassic acid, asiatic acid, and the glycosylated derivative asiaticoside (**40**), the saponin of asiatic acid, as potential apyrase ligands [275]. The *Schistosoma mansoni* NTPDase (*Sm*NTPDase) isoforms exhibit immune cross-reactivity with potato (*Solanum tuberosum*) apyrase, reflecting their high sequence homology and similarity in active-site architecture [276]. Being easily obtained in high purity, potato apyrase has been used for initial screening of complex samples, including plant extracts, aiming to identify compounds with affinity for *Sm*NTPDases [275,277].

After isolation of **40** from the plant extract, the apyrase inhibitory activity of the triterpenoid saponin was evaluated in vitro [275]. Results showed that **40** was a moderate inhibitor of potato apyrase, reducing enzymatic activity by 33% at 50 μM and 43% at 100 μM [275]. Molecular docking analysis indicated that **40** binds within the active site of potato apyrase, *Sm*NTPDase 1, and *Sm*NTPDase 2 with binding energies of −9.9, −10.0 and −11.3 kcal/mol, respectively, consistent with spontaneous complex formation [275]. The ligand formed hydrogen bonds with the catalytic glutamic acid residue (E145, E201, and E164 from apyrase, *Sm*NTPDase1, and *Sm*NTPDase2, respectively) and additionally interacted with hydrogen-bond donor and acceptor residues within the nucleotide-binding pockets of the enzymes. These interactions suggest a potential inhibitory effect on enzymatic activity. Estimated theoretical inhibition constants (*K*_i_) were 55 nM for potato apyrase, 47 nM for *Sm*NTPDase 1, and 5 nM for *Sm*NTPDase 2 [275]. The in vivo studies in mice harboring a patent *S. mansoni* infection showed that a single oral dose of **40** (400 mg/kg) was enough to significantly reduce total worm burden and fecal egg load by 65.4% and 67.7%, respectively [275]. Despite the potential inhibition of *Sm*NTPDases by **40**, additional mechanisms may contribute to its antischistosomal activity, including alteration in membrane permeability and tegument disruption. After oral administration, **40** undergoes biotransformation, being deglycosylated by gut microbiota to yield its aglycone asiatic acid, which can subsequently be hydroxylated to form madecassic acid. Both metabolites were also identified as potential apyrase inhibitors in the *C. erecta* screening assay, alongside **40** [275]. Taken together, these findings support further investigation of *Sm*NTPDase inhibitors, including asiatic and madecassic acids, as potential antischistosomal agents. The antischistosomal activity of plant-derived triterpenoid saponins is summarized in Table 6.

Although the available studies provide valuable insights into the antischistosomal potential of plant-derived terpenoids, the current literature does not yet support a comprehensive or cross-class Structure–Activity Relationship (SAR) analysis. Most reports evaluate individual compounds or small sets of analogues under heterogeneous experimental conditions, limiting direct comparison. Nevertheless, some local trends can be observed. Among monoterpenoids and sesquiterpenoids, increased oxidation (e.g., presence of ketone or epoxide groups), electrophilic centers capable of reacting with parasite proteins, and specific stereochemical configurations have been associated with enhanced activity in certain subclasses. For diterpenoids and triterpenoids, structural rigidity and the presence of functional groups that modulate lipophilicity appear to influence parasite penetration and overall potency. These observations, while informative, remain fragmentary and highly dependent on the specific compounds tested. A more robust SAR framework will require systematic evaluation of structurally related series under standardized assay conditions.

## 5. Mechanisms of Action of Terpenes Against Schistosoma

The mechanism(s) of action and molecular target(s) of antischistosomal plant-derived terpenes remain unclear due to the broad pleiotropic effects exerted by these compounds, similarly to other classes of natural products [104,106,110]. Terpenoids are known to perturb lipid membranes, including the schistosomal tegument, induce oxidative stress, disrupt ion homeostasis, and impair redox balance and energy metabolism. The ability of the lipophilic terpenoids to penetrate across cellular membranes, enabling interactions with intracellular macromolecules and/or organelles, likely contributes to their broad spectrum of activity and ability to disrupt critical processes in the parasite [140,197,222,228]. Many terpenoids have additional immunomodulatory and/or anti-inflammatory properties that contribute to their anti-fibrotic, disease modifying effects by suppressing HSC activation, inhibiting inflammatory signaling, and reducing collagen deposition [160,164,165]. These effects are particularly relevant since fibrosis, not worm burden, is the main cause of morbidity in chronic schistosomiasis [3,8]. The chemical diversity of antischistosomal terpenoids and their multifaceted mode of action, which do not rely on a single preferential target but instead act on multiple parasite systems simultaneously, enhance their therapeutic potential and reduce the likelihood of resistance, supporting the continued interest in medicinal plants as sources of new antischistosomal agents [72,197].

The membrane-directed activity of terpenoids is probably the most consistently reported mechanism underlying their antiparasitic as well as antimicrobial properties. The hydrophobic skeleton of terpenoids allows them to embed within the parasite’s membranes, perturbing membrane structure and increasing permeability, which disrupts ion balance and leads to loss of membrane potential [278,279]. Collapse of membrane integrity, impairment of membrane-associated proteins, and osmotic imbalance further contribute to swelling and eventual lysis, ultimately leading to parasite death. Terpenoid-induced alterations in lipid membranes are concentration-dependent and correlate with the terpene membrane-water partition coefficient [280]. Nerolidol (**19**, discussed in Section 4.2), with a high octanol–water partition coefficient (log*P*_o/w_ = 4.31), causes major reorganization in the lipid bilayer, and at high concentration, lipid extraction and leakage of the cytoplasmic content [280]. Terpenoids have been shown to interact with lipid bilayers across many organisms, including bacteria [124,125,126,132,133,200,205], protozoa [136,137,138,139,176,178,181,199,215,217,218], and helminths [151,152,207], altering membrane organization, fluidity, and protein–lipid interactions, with profound effects in membrane dynamics and behavior of embedded proteins [214,215,216]. Disruption of tegument integrity is a mechanism frequently highlighted in studies of medicinal plant extracts and essential oils with antischistosomal properties [109,112,142,144,150,156,172,180,223,235].

Damage to the tegument is often observed in schistosomes following exposure to terpenoids, and a correlation has been found between the severity of tegumental damage and schistosomicidal activity [155,186,222,230]. The schistosome tegument is a syncytial, membrane-rich and metabolically active surface, which is in direct contact with the host microenvironment [281]. Breaching of the tegument leads to impaired nutrient uptake and osmoregulation disruption, increasing susceptibility to further biochemical stress while the exposed antigens on the surface enhance host immune recognition, thus compromising the parasite’s survival. Terpenoids can induce structural, morphological, and ultrastructural alterations in the schistosome tegument, producing several types of lesions depending on concentration and exposure time as well as on *Schistosoma* species, strain, developmental stage, and eventually sex [194]. These include blebbing and surface vesiculation, a hallmark of membrane-destabilizing compounds in which early tegumental stress produces blister-like protrusions as the syncytial surface begins to detach from underlying tissues [158,222,231,264,273]. More intense injury leads to erosion and peeling of the outer membrane, with sloughing of the apical layers and exposure of the basal lamina, which increases vulnerability to host immune factors. Terpenoids also compromise the spine–tubercle complex, causing shortening, blunting, or complete loss of spines, while tubercles may flatten, collapse, or lose their characteristic contours as the supporting syncytium deteriorates [158,222,231,264,273]. At the ultrastructural level, terpenoids induce swelling or degeneration of mitochondria and membranous bodies, and promote cytoplasmic vacuolation, reflecting metabolic stress and membrane injury. Severe lesions manifest as tegumental fissures, cracks, pits, and ulcer-like defects, indicating breakdown of the protective barrier and contributing to impaired osmoregulation and heightened susceptibility to immune attack. As damage progresses, subtegumental tissues become exposed, revealing near-complete loss of the protective surface and representing a terminal stage of tegumental injury [158,222,231,264,273]. These combined structural, morphological, and ultrastructural alterations illustrate the broad vulnerability of the schistosome tegument to membrane-active and pleiotropic terpenoids.

Terpenoids, in addition to their ability to permeate cellular membranes, can interact with multiple protein receptors and enzymes, interfere with distinct signaling pathways, and modulate host immune responses [140,222,228]. The antiparasitic activity of STLs (discussed in Section 4.2), such as parthenolide (**11**) and vernodalin (**13**) and goyazensolide (**14**), is often attributed to their electrophilic α,β-unsaturated lactone moiety, which undergoes Michael-type addition with biological nucleophiles, particularly thiol groups in parasite proteins [181,185,186,187,192,194]. This reaction can irreversibly alkylate critical enzymes and transcription factors involved in gene regulation, protein synthesis, and cellular metabolism. However, the same reactive functionality also underlies the cytotoxicity frequently associated with these compounds.

Terpenoid-induced oxidative stress creates a hostile environment for the parasite, depleting glutathione and inhibiting several enzymes essential for schistosome antioxidant defense, including the thioredoxin–glutathione reductase (TGR) system, glutathione-S-transferase (GST), glutathione peroxidase (GPx), superoxide dismutase (SOD), and peroxiredoxins (Prx) [72,85]. Notably, unlike mammals, schistosomes rely on a single enzyme, TGR, to regenerate both thioredoxin and glutathione, making TGR indispensable for maintaining redox balance [72,85]. Adult worms depend heavily on peroxiredoxins and TGR to withstand the oxidative environment of the mammalian bloodstream [72,85]. Impairment of the oxidative stress response results in tegumental lesions and damage to subtegumental tissues and organelles, ultimately reducing parasite fertility [72].

Reduction in oviposition by phytol (**21**; discussed in Section 4.3) has been postulated to be associated with inhibition of 3-hydroxy-3-methylglutaryl-coenzyme A (HMG-CoA) reductase, consistent with the cholesterol-lowering properties of this diterpenoid [228]. The *S. mansoni* HMG-CoA reductase (*Sm*HMGR), a key enzyme in the parasite’s mevalonate pathway and crucial for producing non-sterol lipids that stimulate egg production, is essential for parasite survival and reproduction [282]. Statin inhibition of *Sm*HMGR has been shown to reduce egg laying, induce caspase activation and apoptosis, and ultimately lead to parasite death, effects that can be prevented by supplementation with excess mevalonate [283].

Terpenoids can also interfere with the energy metabolism and glucose uptake of schistosomes, which rely heavily on host-derived nutrients for survival. These effects may lead to starvation of worms and insufficient energy supply for growth, pairing, maturation, and fecundity [72,284]. Glycolytic enzymes, including phosphofructokinase (PFK), enolase (ENO), and pyruvate kinase (PK), show reduced activity when exposed to certain terpenes, and oxygenated terpenoids have been reported to disrupt components of the mitochondrial electron transport chain, impairing ATP synthesis [72,284]. The schistosome glucose transporter proteins SGTP4, located on the apical tegumental membrane in direct contact with host blood, and SGTP1, located on the basal membrane of the tegument and internal tissues, are essential for glucose uptake; their inhibition compromises parasite feeding and survival [285]. *S. mansoni* cathepsin B1 (SmCB1), a gut-associated cysteine protease required for host hemoglobin digestion and thus for nutrient acquisition supporting worm growth, development, and reproduction, has also emerged as a promising drug target for schistosomiasis due to structural differences in its active site compared with human cathepsin B, which enables selective inhibition [286].

The acetylcholinesterase (AChE) enzyme located in the schistosome tegument and neuromusculature that regulates cholinergic signaling by terminating acetylcholine (ACh) activity represents a potential drug target. Many plant terpenoids, including citral (**3**), carvacrol (**4**), and thymol, are known AChE inhibitors [287]. Neuromuscular blockade is also the primary mechanism of action of several anthelmintic drugs, such as metrifonate, an AChE inhibitor, and levamisole or pyrantel, which act as agonists of nicotinic acetylcholine receptors (nAChRs), the excitatory ligand-gated cation channels that mediate depolarization and contraction of muscle cells [287]. However, schistosomes possess acetylcholine-gated chloride channels (ACCs), inhibitory cholinergic receptors unique to flatworms (cestodes and trematodes) and evolutionary distinct from those of nematodes [288]. These ACCs are localized to regions of the peripheral nervous system that innervate the body wall muscles, the nerve plexuses of the suckers, and the tegumental surface, with particularly high expression in the male tubercles [288]. Inhibition of schistosomal AChE leads to ACh accumulation, persistent activation of ACCs, and sustained hyperpolarization, resulting in muscle relaxation and flaccid paralysis [287,288]. This contrasts sharply with the excitatory, depolarizing role of ACh in mammalian and nematode neuromuscular systems [287].

The *S. mansoni* sarco/endoplasmic reticulum Ca^2+^-ATPase (*Sm*SERCA), encoded by the SMA1 and SMA2 genes, has also been discussed as a potential target of antischistosomal terpenoids, namely the antimalarial artemisinin-based STLs [289]. Molecular docking studies revealed that AM binds to the thapsigargin-binding cavity (a well-conserved region in parasite SERCAs) in both *Pf*ATP6 and *Sm*SERCA, in either the calcium-bound or calcium-free conformations, with stronger interactions observed for the calcium-free state [289,290]. Thapsigargin, itself an STL and a highly specific SERCA inhibitor, binds the enzyme in the calcium-free form and inhibits it by preventing the conformational change required for pump cycling. The preference of both thapsigargin and AM for the calcium-free state has been attributed to an allosteric change induced by the absence of calcium, which exposes the transmembrane binding pocket to the drug [289]. Thapsigargin antagonizes the parasiticidal activity of ARTs by competing for the same binding pocket [289].

Inhibition of *Sm*SERCA leads to cytosolic Ca^2+^ overload, resulting in sustained muscle contraction and spastic paralysis, and contributes to tegumental destabilization. Impaired motility and tegumental damage are effects commonly observed with antischistosomal terpenoids. Elevated Ca^2+^ levels further disrupt mitochondrial membrane potential, causing mitochondrial dysfunction, increased ROS production, and depletion of the ATP pool. Upregulation of *Sm*SERCA has been reported in *S. mansoni* worms with reduced sensitivity to PZQ [291]. Although antischistosomal terpenoids can induce paralysis through several pathways, the most common outcome is flaccid paralysis, typically resulting from membrane disruption that collapses ion gradients, metabolic stress that reduces the ATP pool, or inhibitory cholinergic effects.

Antischistosomal terpenoids can also act by modulating host immune responses, an aspect increasingly recognized as part of their overall mode of action. The *S. mansoni* ATP diphosphohydrolase (apyrase) *Sm*ATPDase1 is a tegumental ecto-nucleotidase involved in nucleotide metabolism that hydrolyzes extracellular ATP and ADP to AMP, contributing to tegumental homeostasis by regulating purinergic signaling at the host–parasite interface [274]. Enzymatic degradation of host-released ATP, a key danger signal, reduces inflammation and neutrophil activation, enabling the parasite to evade immune detection. Recently, the triterpenoids madecassic acid, asiatic acid and the related saponin asiaticoside (**40**) were identified as potential apyrase inhibitors in a *C. erecta*-based screening assay [275], as discussed in Section 4.5.

Computational approaches may contribute to clarifying the mechanisms of action of antischistosomal terpenoids. In silico methods, such as molecular docking, molecular dynamics simulations, and virtual screening, help predict terpene–protein interactions, identify plausible molecular targets, and assess the stability and specificity of these interactions within essential schistosome pathways [173,292]. Recently, computer-aided drug design (CADD) studies using natural plant-derived STLs from the Asteraceae family as sources of novel bioactive scaffolds for NTDs, including schistosomiasis, have helped elucidate their mechanisms of action, identify key pharmacophoric features, establish highly predictive QSAR models, and uncover new lead molecules [173].

Information from computational studies can be complemented by omics technologies. Genomic, transcriptomic, proteomic, and metabolomic datasets enable the identification of transcriptional, proteomic, and metabolic changes induced by terpenoid exposure, thereby highlighting the pathways and cellular processes most affected in the parasite [293,294,295,296,297,298]. SEM phenotypic analysis of a more potent derivative of sclareol (**22**; discussed in Section 4.3), together with untargeted metabolomics data, suggests that this compound affects membrane lipid homeostasis by interfering with arachidonic acid metabolism [231]. Comparative transcriptomic and proteomic analysis may reveal differential gene expression across developmental stages and sexes of *Schistosoma* species, as well as the localization of putative secretory and membrane antigens, enzymes, and other gene products on the adult tegument and eggshell, many of which display genetic polymorphisms [295]. Identifying differentially expressed proteins between males and females may elucidate critical signaling pathways underlying sexual maturation and egg production, and targeting of these sex-biased proteins may provide potential chemotherapeutic interventions against schistosomiasis [293].

Functional genomics approaches, such as clustered regularly interspaced short palindromic repeats (CRISPR)-based perturbations [299,300] and RNA interference (RNAi) screening [301,302], help validate and prioritize targets emerging from omics datasets. RNAi screening, a loss-of-function strategy that systematically knocks down genes to observe phenotypes such as viability, motility, or structural integrity, has been used to identify therapeutic targets in *Schistosoma* species, including genes affecting neuromuscular function, tissue integrity, and parasite survival [301,302]. A combination of bioinformatics, cheminformatics, functional genomics, and whole-organism approaches has been successfully employed for the identification of epigenetic druggable targets in *S. mansoni* [292].

Furthermore, artificial intelligence (AI) can accelerate the discovery and development of plant-derived antischistosomal terpenoids by linking chemical diversity to biological relevance and predicted interactions with observable phenotypes [4,303]. Machine-learning (ML) models can screen vast libraries of natural terpenoids and predict which structural features are most likely to inhibit key *Schistosoma* targets, reducing the need for time-consuming, bench-based experimentation, a strategy supported by recent ML frameworks applied to natural products with antischistosomal activity [304,305]. AI-driven QSAR modeling, molecular docking refinement, and generative chemistry can highlight promising analogues, optimize lead compounds, and anticipate off-target effects or toxicity early in development [306,307]. When integrated with omics and functional-genomics datasets, AI can also uncover parasite pathways modulated by terpenoids, identify synergistic combinations, and reveal biomarkers of response, in line with ML methods that map parasite mechanisms and potential targets [305]. Together, these approaches create a more efficient, data-driven pipeline for transforming plant-derived terpenoids into viable antischistosomal candidates.

## 6. Nano-Enabled Delivery Systems for Antischistosomal Terpenes

A major drawback of antischistosomal terpenes is their low oral bioavailability usually associated with their poor aqueous solubility, instability in the gastrointestinal tract or rapid hepatic clearance. These limitations often explain why compounds with potent in vitro schistosomicidal activity fail to achieve comparable therapeutic effects in vivo. Nano-enabled drug delivery systems (DDSs) are promising strategies to address the challenges of schistosomiasis therapy with terpenoids by enhancing their solubility, bioavailability, and stability, contributing to improving the pharmacokinetic profile and reducing toxic side effects. Moreover, nanocarriers allow targeted and controlled delivery, which can improve terpenoid’s efficacy and potency [308,309,310].

Nano-enabled delivery of plant-derived schistosomicidal terpenoids can markedly enhance their therapeutic performance not only by increasing bioavailability, but also by improving accumulation in infected tissues, and supporting both efficient and sustained uptake by the parasites. Because schistosomula remain in the liver for roughly 2–3 weeks during their development, nanoformulations with sustained-release properties are especially valuable as they can maintain effective drug levels throughout this hepatic phase and thereby contribute strongly to prophylaxis and early-stage intervention [308]. Nevertheless, few plant-derived schistosomicidal terpenoids have been incorporated into nanoparticulate delivery systems [308].

Among the available nanoparticulate delivery systems, the ones most frequently employed for the delivery of antiparasitic drugs and terpenoids are mainly lipid-based, such as liposomes, solid lipid nanoparticles (SLNs), nanostructured lipid carriers (NLCs), nanoemulsions (NEs), and self-emulsifying drug delivery systems (SEDDS), which spontaneous form micro/nanoemulsions in the gastrointestinal tract [308,311]. These systems can enhance drug exposure, tegument penetration, parasite accumulation, and also host-directed immunomodulatory effects, reducing pathology, fibrosis, and chronic inflammation, key drivers of morbidity in schistosomiasis [308,311].

Liposomes used in drug delivery are usually small unilamellar vesicles (SUVs) composed of a biocompatible phospholipid bilayer membrane that can encapsulate both hydrophobic and hydrophilic compounds in the lipidic layers and aqueous core, respectively [311,312]. However, liposomes have limited stability and often require surface functionalization with poly(ethylene glycol) (PEG) to reduce opsonization and macrophage uptake, leading to stealth liposomes and prolonging circulation in the bloodstream [310,311,312]. Liposomes have been used for delivery of PZQ [313,314] and amphotericin B (AmB) [315,316], and liposomal formulations of AmB are commercially available for treatment of leishmaniasis and fungal infections [315]. PZQ incorporated into liposomes showed enhanced antischistosomal activity in *S. mansoni*-infected mice [313,314]. Liposomes loaded with citral (**3**) or carvacrol (**4**), monoterpenoids discussed in Section 4.1, have also been prepared for improved antifungal activity [317], but the effects on their antischistosomal activity have not been evaluated. However, the liposomal formulation significantly reduced the cytotoxicity of **4** toward macrophages (RAW 264.7 cell line) [317], supporting the use of nanocarriers to improve the safety profile of schistosomicidal terpenoids.

SLNs, made from biocompatible lipids that remain solid at room and body temperature, improve delivery of hydrophobic drugs and provide sustained and controlled release, but sometimes with initial burst effect [311,312]. NLCs are second-generation SLNs made of a blend of solid and liquid lipids (oils) stabilized by a surfactant layer, providing higher loading capacity, enhanced stability during storage, and more stable and predictable controlled and sustained release properties [311,312]. NEs are metastable colloidal dispersions of oil-in-water (O/W) or water-in-oil (W/O) with droplet sizes usually in the range 20–200 nm, stabilized by surfactants/cosurfactants that reduce interfacial tension [311,312,318]. O/W NEs are highly attractive oral delivery systems for lipophilic terpenoids, which become encapsulated within nanosized oil droplets that protect them from degradation and enhance their solubility, absorption, and membrane permeation, thereby improving their overall bioavailability [309,315,318].

The antimalarial STLs AM and AS (repositioned for schistosomiasis) have been encapsulated into SLNs [319,320], NLCs [321,322], and NEs [323,324] for improved bioavailability and enhanced antiprotozoal activity. AM-loaded NLCs were also tested in vitro against *Leishmania* [325], but no formulation has been evaluated against *Schistosoma* parasites yet. Similarly, ursolic acid (**32**; discussed in Section 4.4) and phytol (**21**; discussed in Section 4.3) were incorporated into chitosan-coated NLC [326] and soybean oil NE [327] respectively, for improved leishmanicidal activity. An olive oil NE loaded with betulinic acid (**33**; discussed in Section 4.4) was found to increase 4.4-fold the relative oral bioavailability of the triterpenoid and to enhance its hepatoprotective properties [328]. Meanwhile, SEDDS formulations of oleanolic acid (**31**; discussed in Section 4.4) were developed for improved oral bioavailability. Both self-microemulsifying (SMEDDS) [329] and self-nanoemulsifying drug delivery system (SNEDDS) formulations [330], with droplet sizes of 49.7 and 38.4 nm, respectively, allowed for in vitro sustained release and significantly enhanced relative bioavailability of **31** following oral administration to rats (50 mg/kg) when compared with the commercial tablet. These studies illustrate the potential use of SEDDS to improve oral bioavailability of poorly water-soluble antischistosomal terpenoids like **31**.

Carvacrol (**4**), a phenolic monoterpene commonly found in the essential oils of many plants, especially oregano (*Origanum vulgare*) and thyme (*Thymus vulgaris*) from the mint family Lamiaceae, has shown antimicrobial, antioxidant, anti-inflammatory, acaricidal, and anthelmintic properties [152,331,332], including antischistosomal [150] (discussed in Section 4.1). However, **4** exhibits slow intestinal absorption following oral administration, with over 30% remaining in the gastrointestinal tract [331,332], which limits its activity against blood-dwelling parasites such as schistosomes. Xavier et al. prepared a **4**-loaded O/W NE (carvacrol nanoemulsion, CVNE) by ultrasonic emulsification, using a formulation composed of 3% (*w*/*w*) carvacrol oil, 9% (*w*/*w*) surfactant mixture of hydrophilic polysorbate 80 (Tween 80) and hydrophobic sorbitan monostearate 80 (Span 80) surfactants with hydrophilic–lipophilic balance (HLB) 11, and 88% (*w*/*w*) water [333]. The CVNE displayed a monodisperse profile with a narrow size distribution, presenting an average droplet diameter of 124 ± 0.80 nm and a polydispersity index (PDI) of 0.20 ± 0.01. The nanoformulation had a pH of 5.4 and a zeta potential of −26.4 mV, consistent with an electrostatically stabilized NE, and showed no visible signs of creaming or phase separation over 90 days [333]. The in vitro evaluation of the antischistosomal activity of CVNE and free **4** against adult *S. mansoni* yielded EC_50_ values of 62.37 μM and 56.81 μM, respectively, while the blank NE was inactive [333]. The slightly higher EC_50_ observed for CVNE probably reflects its sustained-release profile, in contrast to free **4**, which is immediately available at higher local concentrations at the parasite surface. Both the drug-loaded and blank NEs showed no in vitro cytotoxicity toward mammalian cells at the highest concentration tested (200 μM) [333]. A single oral dose (200 mg/kg) of CVNE administered to mice harboring patent or prepatent *S. mansoni* infections was more effective than free **4** in reducing worm burden and egg production, whereas the blank NE was inactive [333]. The higher in vivo antischistosomal activity of CVNE is likely attributable to the improved aqueous solubility of **4** and its enhanced absorption across the intestinal epithelium when dispersed in nanodroplets. Moreover, CVNE was more effective than PZQ in prepatent infection, reducing worm burden and fecal egg load by 86.4% and 90.1%, respectively, compared with 29.2% and 31.9% for the reference drug. Free **4** showed activity comparable to PZQ, reducing worm and egg burdens by 30.3% and 30.1%, respectively [333].

The authors suggested that the previously described antimicrobial properties of CVNE [334] may have contributed to its antischistosomal activity by reducing the host gut microbiota, since depletion of gut microbiota by antibiotics has been shown to ameliorate intestinal pathological injury in *Schistosoma japonicum*-infected mice [160]. This microbiota-dependent modulation of the immune response likely helped limit excessive intestinal inflammation and tissue damage by decreasing the levels of IL-4, IL-5, and IL-13 while promoting IL-10 and TGF-β production in infected mice [160]. However, no further studies were conducted regarding the mechanism of action of CVNE in schistosomiasis.

A similar NE formulation was prepared by de Souza et al. with the aim of improving the in vivo efficacy of **5** [335]. The high lipophilicity of **5** contributes to its low oral absorption by limiting aqueous solubility, and nanoemulsification represents an effective strategy to enhance its oral bioavailability. The **5**-loaded nanoemulsion (CANE) showed good stability, with a zeta potential below −28 mV, a pH of approximately 4.0, and an average droplet size of 100 nm with a PDI below 0.3, indicating a homogeneous population. CANE exhibited in vitro activity against *S. mansoni* adult worms (IC_50_ = 28.4 μM), superior to that of free **5** (IC_50_ = 51.8 μM), without observable cytotoxic effects in human or animal cell lines (CC_50_ > 500 μM), while the blank nanoformulation was inactive [335]. A single oral dose (200 mg/kg) of CANE administered to mice harboring either adult (patent infection) or juvenile (prepatent infection) *S. mansoni* was more effective than the parent compound in reducing worm burden and egg production [335]. The reduction in fecal egg load was attributed to the decrease in adult parasite numbers following CANE treatment [335].

The efficacy of CANE in prepatent infection was also superior to PZQ, reducing worm burden and fecal egg load by 42.8% and 44.6%, compared with 22.9% and 24.5% for free **5** and 27.6% and 29.6% for the reference drug [335]. A similar pattern was reported for **4**-loaded NEs in a mouse model of schistosomiasis [333]. In contrast, CANE was less effective than PZQ against patent infection, reducing worm burden and fecal egg load by 36.5% and 59.3%, compared with 29.4% and 48.3% for free **5** and 87.4% and 92.7% for PZQ [335].

Alternatively, polymer-based nanocarriers have also been employed for antiparasitic drug and terpenoid delivery, mostly polymeric NPs made of biocompatible and biodegradable polymers of synthetic or biological origin, such as poly(lactic-co-glycolic acid) (PLGA), PEG, chitosan, and alginate NPs [312,336]. Polymeric NPs offer a versatile drug delivery platform since their size, stability, release profile, and surface chemistry can be precisely tuned while maintaining biocompatibility and low toxicity [310,336]. Surface functionalization with appropriate ligands or antibodies enables selective targeting and allows the design of smart nanocarriers capable of releasing concentrated therapeutic payloads on demand in response to internal or external stimuli [310,336]. AS-loaded PLGA NPs [337] and betulinic acid (**33**)-loaded chitosan NPs [338] showed enhanced antiprotozoal activity in vivo in mouse models of malaria and *Leishmania*, respectively. This finding suggests that the antischistosomal activity of **33** (discussed in Section 4.4) can be improved by using nanocarriers, such as polymeric NPs. Parthenolide (**11**; discussed in Section 4.2) has also been encapsulated in PLGA NPs to explore the cytotoxic potential of this STL in leukemic cell lines [339]. Nanoencapsulation of carvacryl acetate (**5**; discussed in Section 4.1) in chitosan/chichá gum NPs [340] (or chitosan/arabic gum NPs [341]) reduced the toxicity of the phenolic monoterpenoid in mice (2609 mg/kg vs. 1544.5 mg/kg for free **5**). However, it did not improve the anthelmintic activity against sheep gastrointestinal nematodes after oral administration when compared with free **5** (both at 250 mg/kg). The reduction in toxicity was attributed to the sustained release of **5** from the chitosan NPs, preventing potentially toxic peak plasma concentrations [340,341].

Cyclodextrin (CD) complexation is another nanotechnological strategy for terpenoid delivery that enhances solubility, stability, and bioavailability of highly lipophilic phytochemicals while operating at the nanoscale through inclusion-complex formation. CDs are cyclic oligosaccharides composed of α-D-glucopyranose units linked by α-1,4-glycosidic bonds and arranged in a truncated cone shape, with a hydrophobic internal cavity and a hydrophilic outer surface [188,342]. The number of glucopyranose units defines the main natural types of CDs and the size of their cavity, with α-, β-, and γ-CDs possessing 6, 7, and 8 glucopyranose units, respectively, and cavity sizes of 0.45–0.53, 0.60–0.65, and 0.75–0.83 nm [188,342]. By encapsulating terpenoids within their hydrophobic cavity, CDs protect them from oxidation and chemoenzymatic degradation while enabling their dispersion in aqueous environments, thus improving absorption. Moreover, CD complexation supports combination with other nanocarriers such as liposomes, polymeric NPs, or NEs. Evaluation of drug-in-CD-in-liposomes as an encapsulating system for nerolidol (**19**; discussed in Section 4.2) showed enhanced photostability and prolonged release compared with conventional liposomes [343].

Among the available CDs, β-cyclodextrin (βCD) provides strong inclusion capacity but has limited aqueous solubility and cytotoxicity at high concentrations whereas 2-hydroxypropyl-β-cyclodextrin (HPβCD), a modified βCD, offers enhanced water solubility, lower toxicity, and more flexible complexation behavior, making it more suitable for biological applications, such as formulating sensitive terpenoids intended for oral delivery [188,342].

Queiroz et al. developed cnicin (**12**) inclusion complexes with βCD and HPβCD, prepared by coprecipitation, aiming to improve the aqueous solubility and oral bioavailability of this antischistosomal STL [188,342]. (discussed in Section 4.2). Contrary to free **12**, the **12**/βCD and **12**/HPβCD inclusion complexes were inactive against both male and female adult *S. mansoni* after 24 h of incubation at 50 μM, the highest concentration tested [188]. This may be due to controlled release from CD complexes requiring longer time for dissociation and release of free **12**, the active form [188]. Nevertheless, complexation with HPβCD decreased cytotoxic potential of **12** toward mammalian cells, presumably by masking its α-methylene-γ-lactone moiety, thus improving selectivity [188]. Further in vivo evaluation of the **12**/CD inclusion complexes was performed in a chronic murine model of schistosomiasis [188]. Mice harboring adult *S. mansoni* were treated with three daily doses of **12** or its CD complexes, administered orally (100 mg/kg) or intraperitoneally (10 mg/kg). Similarly to free **12**, oral treatment with **12**/βCD produced no significant reduction in worm burden while i.p. administration decreased total worm load by 48.1% compared with the untreated control group. Both oral and i.p. treatments with either **12**/βCD or free **12** caused adult worm pairs to separate into individual males and females [188]. Likewise, no significant effects on the number of OPG in feces were observed after oral or i.p. administration of either **12**/βCD or free **12** [188]. In contrast, **12**/HPβCD displayed the highest therapeutic efficacy, reducing total worm burden by 56.8% and OPG by 70.5% after oral administration, and by 66.7% and 97.9%, respectively, after i.p. administration. The higher in vivo efficacy of the **12**/HPβCD complex may be attributed to the improved aqueous solubility of **12**, as the hydroxyl and hydroxypropyl groups of HPβCD enhance water solubility compared with βCD [188].

Blank βCD or HPβCD were inactive against *S. mansoni* both in vitro and in vivo, supporting the conclusion that the antischistosomal activity of the **12**/CD complexes results from the delivery of encapsulated **12** to parasite cells [188]. This interpretation was further supported by in vivo permeability studies using Nile red-CD inclusion complexes. Fluorescence microscopy of *S. mansoni* recovered from mice after i.p. administration of Nile red-HPβCD showed that the complex reached the parasite in vivo and penetrated the tegument of both male and female adult worms, enabling targeted drug delivery [188].

Thus, nanocarrier-based delivery systems offer a compelling strategy to unlock the full therapeutic potential of terpenoids against schistosomiasis. By enhancing solubility, stability, and bioavailability, these nanoparticulate systems overcome the major pharmacokinetic limitations that usually restrict the clinical use of plant-derived schistosomicidal agents. Their ability to promote targeted accumulation in infected tissues, protect labile compounds, and provide sustained release matches the parasite’s developmental dynamics, particularly the prolonged hepatic phase of schistosomula [308]. Collectively, these advantages position nanocarriers as a promising platform for improving treatment efficacy, reducing dosing frequency, and supporting prophylactic interventions, underscoring their relevance for future development of terpenoid-based antischistosomal therapies.

Nevertheless, despite providing powerful strategies to overcome key pharmacokinetic constraints, it is important to recognize that these approaches cannot compensate for intrinsic limitations of the parent molecule. In particular, nanoformulation cannot rescue compounds that lack sufficient target affinity, display weak intrinsic potency, or exhibit unacceptable toxicity profiles at pharmacologically relevant concentrations. Therefore, a clear distinction must be made between terpenoids whose in vivo failure is primarily driven by delivery and pharmacokinetic barriers (e.g., nerolidol or carvacryl acetate), and those that require further structural optimization to enhance potency, selectivity, or safety (such as certain sesquiterpene lactones with reactive electrophilic moieties). In this context, nano-delivery should be viewed as a complementary enabling strategy within the drug development pipeline, rather than a universal solution.

## 7. Conclusions

The remarkable structural diversity, broad biological activities, and abundance of terpene compounds in medicinal plants used in endemic regions highlight their pharmacological value, while their synergistic interaction with PZQ supports their inclusion in combination therapies to enhance efficacy and curb resistance. Furthermore, their capacity to disrupt key molecular and physiological processes across multiple stages of the *Schistosoma* life cycle—including tegumental damage, oxidative stress induction, neuromuscular impairment, and immunomodulatory effects—highlights the mechanistic breadth of terpenes and terpenoids as antischistosomal agents.

Plant-derived terpenes, from mono- to triterpenes, and triterpenoid saponins, constitute a diverse reservoir of antischistosomal candidates with complementary mechanisms of action. Monoterpenes and sesquiterpenes show relevant activity but are limited by weak pharmacokinetics and stage-dependent efficacy. Diterpenes display multistage, multi-target effects, including tegumental disruption and reproductive impairment, though their development is constrained by variable potency and bioavailability. Triterpenes, including lupane, cucurbitane, limonoid and lanostane scaffolds, offer moderate to strong activity with additional host-directed benefits but still require structural refinement. Notably, triterpenoid saponins such as hederacolchiside derivatives and asiaticoside exhibit exceptional potency—particularly against early juvenile stages—and exert immunomodulatory and antifibrotic effects, although their extremely low oral bioavailability necessitates advanced delivery strategies. Collectively, these findings underscore the promise of terpenes as multi-mechanistic antischistosomal leads while highlighting the need for innovative formulation approaches to overcome pharmacokinetic barriers and unlock their therapeutic potential.

Nano-enabled formulations provide a compelling solution to these pharmacokinetic limitations. Lipid-based nanocarriers such as nanoemulsions, nanostructured lipid carriers and self-emulsifying drug-delivery systems have demonstrated the ability to enhance solubility, stability, systemic exposure and tissue accumulation, improving in vivo efficacy even against juvenile worms that are poorly responsive to PZQ. Polymeric nanoparticles and cyclodextrin complexes further expand formulation possibilities by enabling controlled release, improved safety profiles and enhanced bioavailability of lipophilic or labile terpenes. These advances suggest that rationally engineered delivery systems may unlock the full therapeutic potential of terpene-based scaffolds and support their integration into combination therapies with PZQ to enhance efficacy and mitigate resistance, taking into account the reservations highlighted in Section 6.

At the same time, schistosomiasis control is being reshaped by complex immunological, ecological and epidemiological dynamics. Chronic helminth exposure, periodic PZQ-mediated immune resetting and declining natural transmission generate hybrid immune states that differ markedly from those in non-endemic populations. A deeper understanding of these real-world immune landscapes—including the influence of co-infections, cumulative exposure and microbiome composition—is essential for refining diagnostics, improving vaccine design and guiding post-MDA surveillance. Advances in parasite genomics, transcriptomics, proteomics and metabolomics have expanded the repertoire of potential therapeutic and diagnostic targets, yet translation remains limited by the scarcity of robust functional genetic tools. The development of reliable somatic and germline transgenesis platforms is therefore critical for validating candidate pathways and accelerating rational intervention design. Mechanistic studies further highlight that morbidity reduction will depend not only on antiparasitic efficacy but also on targeting immunomodulatory circuits, including egg-induced pathology, macrophage polarization and granuloma dynamics.

The search for alternative chemotherapeutic agents has gained renewed urgency, particularly in the context of hybrid *Schistosoma* species and concerns regarding emerging PZQ tolerance. Natural-product-derived chemotypes—including terpenes—are showing promising activity across developmental stages, and their semisynthetic refinement offers opportunities to enhance potency, selectivity and pharmacokinetic performance. In parallel, computational and artificial-intelligence-driven pipelines are transforming early-stage discovery. Virtual screening, molecular docking, QSAR modelling and generative design now enable high-precision prioritization of terpenoid analogues, exploration of vast chemical space and early identification of off-target liabilities. When integrated with omics and functional-genomics datasets, these tools help map drug-modulated pathways, uncover synergistic combinations and identify biomarkers of response. Automated high-throughput phenotypic platforms further accelerate this trajectory by enabling standardized assessment of compound effects on motility, viability, tegumental architecture and stage specificity.

Among the diverse terpenoid scaffolds reviewed, a limited number emerge as particularly promising lead candidates for further development. Notably, nerolidol and related derivatives represent attractive leads due to their broad-spectrum antiparasitic activity, favorable safety profile, and demonstrated in vivo efficacy, although pharmacokinetic optimization remains necessary. Paeoniflorin stands out for its dual antiparasitic and antifibrotic effects, targeting not only parasite burden but also the host-driven pathology that underlies chronic disease. Additionally, triterpenoid saponins such as hederacolchiside A1 and asiaticoside exhibit remarkable potency, including activity against early developmental stages, although their poor oral bioavailability highlights the need for formulation or structural optimization. Together, these examples illustrate that successful translation will require an integrated approach combining potency, selectivity, pharmacokinetics, and disease-modifying capacity.

Nevertheless, pharmacological innovation alone will not achieve elimination. Schistosomiasis remains deeply intertwined with poverty, limited access to healthcare, inadequate sanitation and ecological changes driven by climate instability. Strengthened health-system capacity, sustained political commitment and multisectoral interventions remain indispensable, particularly as climate change reshapes snail habitats and increases human displacement. A One Health perspective—integrating human, animal and environmental health—will be essential for designing resilient, context-specific strategies.

Taken together, these converging lines of evidence indicate that durable schistosomiasis control requires a multidimensional agenda that couples modern molecular and computational tools with strengthened health infrastructures and innovative therapeutic pipelines. Within this integrated framework, terpenes and related natural products emerge not merely as chemical curiosities but as strategically relevant candidates capable of complementing—or ultimately surpassing—the performance of PZQ. Sustained, interdisciplinary effort will be essential to expand the chemotherapeutic arsenal and to advance toward the long-standing goal of schistosomiasis control and eventual elimination.

## Figures and Tables

**Table 1:** Comparative Overview of Human *Schistosoma* species.

*Schistosoma* spp.	Intermediate Host Snail	Geographical Coverage	Egg Morphology; Location	Adult Location	Typical Clinical Patterns
*S. haematobium*	*Bulinus* spp.	Africa, Middle East, Turkey, India, Corsica (France).	(110–170) µm × (40–70) µm; terminal spine; urine.	Vesical venous plexus	GU: terminal dysuria, haematuria; obstructive uropathy/hydronephrosis; keratinizing metaplasia with increased bladder SCC risk; FGS (pain, dyspareunia, subfertility), increased HIV risk; MGD (haematospermia, infertility).
*S. mansoni*	*Biomphalaria* spp.	Africa, Middle East, some Caribbean islands, Brazil, Venezuela and Suriname.	(114–180) µm × (45–70) µm; prominent lateral spine; stool.	Mesenteric venous plexus	GI: chronic abdominal pain/diarrhoea; periportal fibrosis leading to portal hypertension, splenomegaly, varices/bleeding; hepatic function largely preserved.
*S. japonicum*	*Oncomelania* spp.	China, Indonesia, Philippines, Thailand.	(70–100) µm × (55–64) µm; small lateral knob; rounder; stool.	Mesenteric veins (small bowel)	Often more severe HSD (high egg output); higher likelihood of encephalic lesions in neuroschistosomiasis; portal hypertension as in *S. mansoni*.
*S. mekongi*	*Neotricula* spp.	Southeast Asia (Cambodia, Lao People’s Democratic Republic, *Myanmar*, *Thailand*).	(50–80) µm × (40–65) µm; small spine; stool.	Mesenteric veins	Intestinal/hepatosplenic disease in Mekong foci (Laos/Cambodia); periportal fibrosis and portal hypertension may occur.
*S. intercalatum*/*S. guineensis*	*Bulinus* spp.	Central and West Africa.	(158–164) µm × (59–61) µm; terminal spine; central bulge; stool.	Mesenteric veins	Intestinal disease (often mild) in Central Africa; hepatointestinal spectrum; reports of hybridisation with haematobium/guineensis groups

Abbreviations: GI, gastrointestinal disease; GU, genitourinary disease; FGS, female genital schistosomiasis; SCC, squamous cell carcinoma; MGD, male genital disease; HSD, hepatosplenic disease.

**Table 2:** Antischistosomal activity of plant-derived monoterpenoids.

Compound (Source)	Species (Strain), Type of Study	Treatment	Main Findings	Ref.
(+)-(*R*)-Limonene (**1**) (commercially acquired)	*S. mansoni* (BH strain) In vitro	Adult worms (56-day-old) incubated with **1** (43.75 μg/mL) or vehicle (1.6% DMSO) for 120 h. Positive control: PZQ (0.5 μg/mL).	Decreased motor activity at 43.75 μg/mL after 96 h. No lethal effects.	[156]
(+)-(*R*)-Limonene epoxide (**2**) as diastereomeric mixture (synthesized from **1** by epoxidation)	*S. mansoni* (BH strain) In vitro	Adult worm (49-day-old) couples incubated with **2** at 12.5, 25, 50 and 75 μg/mL or vehicle (0.5% DMSO) for 120 h. Positive control: PZQ (10 μg/mL).	Reduced motility and death of all parasites after 120 h at 25 μg/mL or 24 h at 75 μg/mL. No differential sensitivity between male and female worms. CLSM revealed tegumental destruction associated with **2**-mediated schistosomicidal activity.	[140]
Citral (**3**) (commercially acquired)	*S. mansoni* (LE strain) In vitro	Adult (42-day-old) worm couples incubated with **3** at 5, 25, 50 and 100 μM or vehicle (0.1% DMSO) for 48 h. Positive control: PZQ (10 μg/mL).	Reduced worm viability by more than 75% after 24 h of incubation at 50 and 100 μg/mL. Sublethal concentrations (5 μg/mL) significantly reduced oviposition by approx. 90% after 48 h exposure. Low toxicity at 50 μg/mL (GI% = 64.2 ± 1.6), moderate at 100 μg/mL (GI% = 73.2 ± 8.3) in mouse fibroblast L929 cells.	[150]
Carvacrol (**4**) (commercially acquired)	*S. mansoni* (LE strain) In vitro	Adult (42-day-old) worm couples incubated with **4** at 5, 25, 50 and 100 μM or vehicle (0.1% DMSO) for 48 h. Positive control: PZQ (10 μg/mL)	100% mortality at 100 μg/mL after 2 h of incubation. Sublethal concentrations (5 μg/mL) significantly reduced oviposition by approx. 90% after 48 h exposure. Low toxicity at 50 μg/mL (GI% = 51.7 ± 3.2), moderate at 100 μg/mL (GI% = 73.4 ± 6.2) in mouse fibroblast L929 cells	[150]
Carvacryl acetate (**5**) (Semi-synthetic ester derivative of **4**)	*S. mansoni* (BH strain) In vitro	Adult (49-day-old) worm couples incubated with **5** at 1.562, 3.125, 6.25, 12.5, 25 and 50 μg/mL or vehicle (0.5% DMSO) for 120 h. Positive control: PZQ (10 μg/mL).	At 6.25 μg/mL, **5** reduced parasite mobility and viability after 48 h, but lower doses were inactive. At 25 μg/mL, 100% lethality was achieved after 24 h, with no differential sensitivity between male and female worms. A sublethal dose of 6.25 μg/mL reduced egg production by 90% without affecting worm pairing. CLSM revealed tegumental blebbing and swollen tubercles.	[140]
	*S. mansoni* (BH strain) In vitro	Adult (49-day-old) worms and NTS exposed to **5** at concentrations in the range 0.3–50 μM or vehicle (0.5% DMSO) for 72 h. Positive control: PZQ (0.06–5 μM).	More active against NTS (EC_50_ = 14.58 ± 3.57 μM and EC_90_ = 23.45 ± 4.03 μM, at 72 h) than adult worms (EC_50_ = 42.16 ± 7.64 μM and EC_90_ = 54.08 ± 5.23 μM, at 72 h). PZQ showed EC_50_ and EC_90_ values at 72 h of 0.48 ± 0.15 μM and 0.98 ± 0.13 μM against adult worms and 6.01 ± 1.36 μM and 9.46 ± 2.28 μM against NTS.	[155]
	*S. mansoni* (BH strain) In vivo	Female Swiss mice (3-weeks-old) subcutaneously infected with 80 cercariae and treated with 100, 200 or 400 mg/kg single dose of **5** at 42-days pi or 400 mg/kg at 21-days pi, by oral gavage, or vehicle (2% ethanol in water). Positive control: PZQ (400 mg/kg). Mice were euthanized 56 days pi.	The 200 and 400 mg/kg doses reduced total worm burden by 35.8% and 39.2%, respectively, in patent infection (vs. 90.3% for PZQ). Reduced egg loads in intestines and feces at 400 mg/kg (70.1% and 76.6%, respectively, vs. 92.9% and 93.3% for PZQ). Low efficacy on prepatent infection (31.9% total worm burden reduction), similarly to PZQ (29.5%).	[154]
Dihydrocitronellol (**6**) (commercially acquired)	*S. mansoni* (BH strain) In vitro	Adult (49-day-old) worm couples incubated with **6** at 10, 20, 40, 80, and 160 μM or vehicle (0.5% DMSO) for 120 h. Positive control: PZQ (3 μM).	Reduced motor activity at conc. ≥ 10 μM. 100% lethality at 20 μM within 120 h or at 80 μM within 24 h IC_50_ values of 13.51 and 52.35 μM at 120 h and 24 h, respectively. CLSM revealed extensive, concentration-dependent tegumental damage. A correlation was observed between parasite viability and the severity of tegumental lesions.	[155]
Rotundifolone (**7**) (*Mentha x villosa* Hudson, Lamiaceae)	*S. mansoni* (BH strain) In vitro	Adult (56-day-old) worms incubated with **7** at 0.70, 3.54, 7.09, 71.0, 177.4, 354.8, and 701.0 μg/mL or vehicle (1.6% DMSO) for 120 h. Positive control: PZQ (0.5 μg/mL)	No worm mortality at conc. below 71.0 μg/mL. 100% lethality after 72 h exposure at 71.0 μg/mL, 48 h at 177.4 μg/mL, and 24 h at or above 354.8 μg/mL. SEM revealed dose- and time-dependent morphological alterations, including tegumental lesions and tubercle loss.	[156,158]
	*S. mansoni* (BH strain) In vivo	Female Swiss Webster mice (30-day-old) infected with ± 80 cercariae via tail immersion and treated with **7** (35.9, 70.9 and 141.9 mg/kg/day) or vehicle (suspension in 7% Tween-80 and 3% ethanol), by oral gavage, at 45-days pi, for 5 consecutive days. Positive control: PZQ (200 mg/kg). Mice were euthanized 60 days pi.	Treatment at 35.9, 70.9 and 141.9 mg/kg reduced worm burden by 13.3%, 59.2% and 74.5%, respectively, vs. 95.9% for PZQ. Mice treated with 141.9 mg/kg showed modest reductions in intestinal (22.4%), hepatic (27.3%) and fecal (35.9%) egg counts. Changes in the oogram pattern showed decreased number of immature eggs and increased number of dead eggs.	[143]
Paeoniflorin (**8**) (commercially acquired)	*S. mansoni* (Egyptian strain) In vivo	Swiss albino male CD-1 mice (6–8 weeks old) infected with ± 80 cercariae by s.c. injection and treated with **8** (50 mg/kg/d), PZQ (300 mg/kg/bid) or vehicle (1% CMC), orally, 6 weeks pi, for 30 days. Mice were sacrificed 15 weeks pi.	Reduction in hepatic worm burden (84.3% vs. 92.8% for PZQ) and viable egg %. Reduction in granuloma diameter (66.7% vs. 30.8% for PZQ) and fibrotic areas (86.3% vs. 69.2%). **8** inhibited HSC activation via up-regulation of apoptotic markers (caspase-3 and p53) and down-regulation of fibrotic markers (NF-κB, TGF-β1, α-SMA, and serum IL-13) **8** was more effective than PZQ at ameliorating infection-induced liver fibrosis.	[160]
	*S. mansoni* (Egyptian strain) In vivo	Male BALB/c Swiss albino mice (6–8 weeks old) infected with ± 100 cercariae by tail immersion and treated with **8** (50 mg/kg/day), AM (300 mg/kg/day), PZQ (300 mg/kg/bid) or vehicle (1% CMC), orally, 6 weeks pi, for 30 days. Mice were sacrificed 15 weeks pi.	Reduction in hepatic worm burden (94.3% vs. 83.6% for PZQ and 36.6% for AM). OPG reduction in feces (93.1% vs. 80.0% for PZQ and 38.9% for AM) and liver tissue (93.0% vs. 62.4% for PZQ and 37.5% for AM). **8** was more effective at ameliorating infection-induced fibrosis associated with lower α-SMA expression in pericytes. **8** inhibited angiogenesis by reducing the expression of proangiogenic VEGF, CD34, and vascular PCNA while increasing anti-angiogenic TIMP-2 expression in myofibroblasts, fibroblasts and vascular endothelial cells.	[167]
	*S. japonicum* In vivo	Female BALB/c mice (6–8 weeks old) infected transcutaneously with ~25 cercariae and treated with **8** (30 mg/kg/day) or vehicle (0.5% sodium CMC), orally, on day 12 pi for 30 days, then PZQ (500 mg/kg/day) orally on day 42 pi, for 2 days. Mice were sacrificed on day 102 pi.	No significant effects on worm burden and egg load. Reduced hepatic hydroxyproline by 54.7% Prevented egg-induced deposition of Col I and Col III in the liver. Reduced IL-13 level by 43.0% but increased IL-13Rα2 in liver by 37.7%, which correlated with attenuation of hepatic fibrosis.	[166]
Hinokitiol (**9**) (synthetic powder)	*S. mansoni* (PR strain) In vitro	Approx. 400 cercariae exposed to **9** at 25 and 50 μM or vehicle (water) for 120 min.	TEM revealed ultrastructural changes 15 min after exposure at 25 μg/mL, including cercarial tail loss. Progressive degeneration of tegument and deeper parenchymal tissues with exposure time at 50 μg/mL.	[169]
Linalool (**10**) (commercially acquired)	*S. japonicum* In vitro	100 ± 2 cercariae exposed to **10** at 0.03, 0.06, 0.12, 0.25, 0.5 and 1.0 μg/mL or vehicle (dechlorinated tap water containing 1% (*v*/*v*) ethanol) for 6 h.	LC_50_ = 0.07 μg/mL at 6 h. Damage to tegument and body–tail junction with cercarial tail loss. Additional molluscicidal effects against *Oncomelania hupensis* (LC_50_ = 0.25 μg/mL at 6 h) with damage to gills and hepatopancreas of the snails.	[170]
	*S. japonicum* In vivo	BALB/c mice (8-week-old) infected percutaneously with 30 cercariae and sacrificed 30 min or 45 days after challenge. Cercariae were exposed for 15 min to **10** at 0.1 or 10 μg/mL or dechlorinated tap water containing 1% (*v*/*v*) ethanol prior to challenge.	**10** markedly reduced the recovered schistosomula from mouse skin after 30 min challenge infection. **10** at 0.1 and 1.0 μg/mL decreased worm burden by 27.42% and 60.75%, respectively, and reduced egg counts in liver (by 25.03% and 58.12%) and intestine (by 23.36% and 64.90%) but did not affect fecundity of female adult worms. The cercaricidal activity of **10** can be useful for development of prophylactic topical formulations.	[170]

**Table 3:** Antischistosomal activity of plant-derived sesquiterpenoids.

Compound (Source)	Species (Strain), Type of Study	Treatment	Main Findings	Ref.
Parthenolide (**11**) (*Tanacetum parthenium* L., Asteraceae)	*S. mansoni* (BH strain) In vitro	Adult worm couples incubated with **11** at 6.25, 12.5, 25, 50 and 100 μM or vehicle (0.5% DMSO) for 72 h. Positive control: PZQ (5 μM).	Reduced worm motor activity and separated worm couples into individual males and females. LC_50_ = 9.5 μM at 72 h. 100% mortality after 48 h of incubation at 12.5 μM or 24 h at 25 μM. CLSM revealed extensive tegument damage, which correlated with parasite death.	[186]
Cnicin (**12**) (*Centaurea benedicta*, Asteraceae)	*S. mansoni* (BH strain) In vitro	Adult (49-day-old) worm couples incubated with **12** at 6.25, 12.5, 25 and 50 μM or vehicle (0.5% DMSO) for 48 h. Positive control: PZQ (2 μM).	100% reduction in motor activity and 100% lethality after 24 h at 25 or 50 μM. 100% reduction in motor activity and 100% lethality of female worms after 48 h at 6.25 μM while males were unaffected. CC_50_ = 21.83 ± 0.34 μM (murine peritoneal macrophages) Cytotoxicity to mammalian cells attributed to the reactive α-methylene-γ-lactone moiety of **12**.	[188]
	*S. mansoni* (BH strain) In vivo	Swiss female mice infected percutaneously with 80 cercariae and treated with 3 daily doses of **12** *p.o.* (100 mg/kg) or i.p. (10 mg/kg) or vehicle (2% *v*/*v* ethanol in water) at 49 days pi. Mice were euthanized 63 days pi.	No significant reduction in worm burden after oral administration. Intraperitoneal administration reduced total worm burden by 41.9%. Both treatments induced separation of adult worm couples. Neither treatment reduced fecal OPG. Low oral bioavailability of **12** may account for the reduced efficacy of oral treatment compared with intraperitoneal route.	[188]
Vernodalin (**13**) (*Vernonia amygdalina*, Asteraceae)	*S. japonicum* In vitro	Adult (56-day-old) worm pairs exposed to 20 or 200 ppm of **13**. Positive control: PZQ (2 ppm).	Inhibition of motility and oviposition at 20 ppm (approx. 20 μg/mL) compared with 2 ppm for PZQ.	[192]
	*S. japonicum* In vivo	*S. japonicum*-infected mice treated with 60 or 120 mg/kg, orally.	**13** at 120 mg/kg was lethal. The non-lethal 60 mg/kg dose showed no significant antischistosomal effects.	[192]
Goyazensolide (**14**) (*Eremanthus goyazensis*, Asteraceae)	*S. mansoni* (LE strain) In vitro	Adult (49-day-old) worm couples incubated with **14** at 0.2, 0.4, 0.6, 0.8, 1.0, 2.0, 3.0, 3.5 and 4.0 μg/mL or vehicle (10% DMSO) for 96 h.	Reduced motor activity and egg production at conc. > 0.8 μg/mL. 90% lethality after 24 h at 3.5 μg/mL. Females more susceptible than males. Concentration- and time-dependent tegumental damage.	[194]
α-Humulene (**15**) (commercially acquired)	*S. mansoni* (BH strain) In vitro	Adult (56-day-old) worm couples exposed to **15** at 0, 25, 50, 100 and 200 μg/mL for 72 h. Positive control: PZQ (10 μg/mL).	Dose-dependent schistosomicidal effect (LC_50_ = 149.98 μM). 60% worm mortality at 200 μg/mL after 72 h of incubation. Damage to the tegument and to oral and acetabular suckers. Complete inhibition of excretory system activity only in male worms.	[195]
*trans*-Caryophyllene (**16**) (commercially acquired)	*S. mansoni* (BH strain) In vitro	Adult (56-day-old) worm couples exposed to **16** at 0, 25, 50, 100 and 200 μg/mL for 72 h. Positive control: PZQ (10 μg/mL).	Dose-dependent schistosomicidal effect (LC_50_ = 142.11 μM). 80% worm mortality at 200 μg/mL after 72 h of incubation. Damage to the tegument and to oral and acetabular suckers. Complete inhibition of excretory system activity only in male worms.	[195]
3,6-Epidioxy-bisabola-1,10-dione (**17**) as a diastereomeric mixture (*Drimys brasiliensis*, Winteraceae)	*S. mansoni* (BH strain) In vitro	Adult (49-day-old) worm pairs incubated with **17** at 0.78, 1.56, 3.12, 6.25, 12.5, 25 and 50 μg/mL or vehicle (0.5% DMSO) for 72 h. Positive control: PZQ.	EC_50_ = 4.1 ± 1.2 μM for both male and female worms at 72 h (SI > 48), comparable with PZQ (EC_50_ = 1.1 μM for male and 1.3 μM for female worms). CC_50_ > 200 μM (Vero kidney cells from African green monkey)	[196]
	*S. mansoni* (BH strain) In vivo	Female Swiss mice (3-week-old) subcutaneously infected with 80 cercariae and treated with single dose of **17** (400 mg/kg), PZQ (400 mg/kg) or vehicle, by oral gavage, 42 days pi. Mice were euthanized 56 days pi.	Reduction of total worm burden by 61.2% vs. 85.0% for PZQ. Remarkable egg burden reduction of 98.2% in intestinal tissues and 99.2% in feces, surpassing PZQ efficacy (84.1% and 89.8%, respectively).	[196]
Polygodial (**18**) (*Drimys brasiliensis*, Winteraceae)	*S. mansoni* (BH strain) In vitro	Adult (49-day-old) worm pairs incubated with **18** at 1.56, 3.12, 6.25, 12.5, 25 and 50 μg/mL or vehicle (0.5% DMSO) for 72 h. Positive control: PZQ.	EC_50_ = 9.3 ± 1.7 (female) or 9.6 ± 0.5 μM (male) at 72 h (SI > 21). CC_50_ > 200 μM (Vero kidney cells from African green monkey)	[197]
	*S. mansoni* (BH strain) In vivo	Swiss mice (3-week-old) subcutaneously infected with 80 cercariae and treated with single dose of **17** (400 mg/kg), PZQ (400 mg/kg) or vehicle (water), by oral gavage, 42 days pi. Mice were euthanized 56 days pi.	Reduction of total worm burden by 44.1%. Decrease in egg production of 69.5% in intestinal tissues and 71.8% in feces. Lower efficacy than PZQ, which reduced total worm burden, intestinal and fecal egg loads by 87.4%, 88.9% and 92.5%, respectively.	[197]
Nerolidol (**19**) as a 1:1 mixture of *trans* (**19a**) and *cis* (**19b**) isomers (commercially acquired)	*S. mansoni* (BH strain) In vitro	Adult (49-day-old) coupled worm pairs incubated with **19** at 0, 15.6, 31.2, 62.5, 125 and 250 μM or vehicle (0.5% DMSO) for 120 h. Positive control: PZQ (3 μM).	Separation of all paired adult worms at 15.6 μM. Reduced motor activity of male worms at 31.2 μM and females at 62.5 μM. 100% mortality of male and female worms at 125 μM after 24 h and 48 h exposure, respectively. Male worms more susceptible than females. CLSM revealed tegumental damage which correlated with worm viability.	[222]
	*S. mansoni* (BH strain) In vitro	NTS, juvenile (21-day-old) or adult (49-day-old) worms incubated with **19** at 0, 6.25, 12.5, 25, 50, 100, 200 and 400 μM for 72 h.	LC_50_ values of 117.08, 124.62 and 84.99 μM against schistosomula, juvenile, and adult stages, respectively. **19** is more potent against the adult stage, showing similar activity on schistosomula and juvenile stages.	[224]
	*S. mansoni* (BH strain) In vivo	BALB/c mice (3-week-old) infected with 80 cercariae and treated with 100, 200 or 400 mg/kg single oral dose or vehicle (100 μL corn oil), 49 days pi (patent infection) or 400 mg/kg single oral dose, 21 days pi (prepatent infection). Mice were euthanized 2 weeks post-treatment.	Worm burden reductions of 70.1% at 400 mg/kg and 48.8% at 200 mg/kg. Immature egg burden reductions of 84.6% at 400 mg/kg, 69.7% at 200 mg/kg and 29.9% at 100 mg/kg. Fecal egg load reductions of 75.2% at 400 mg/kg and 48.3% at 200 mg/kg. SEM revealed tegumental damage in both male and female adult worms recovered from treated mice (400 mg/kg, patent infection). Low efficacy in prepatent infection, with reductions in total worm burden of 30.1% and fecal egg load of 26.7%.	[224]
4-Nerolidyl catechol (**20**) (*Pothomorphe umbellata*, Piperaceae)	*S. mansoni* (BH strain) In vitro	Adult worm couples incubated with **20** at 3.12, 6.25, 12.5, 25, 50 and 100 μM or vehicle (0.5% DMSO) for 24 h. Positive control: PZQ (2 uM).	50% mortality at 3.12 μM achieving 100% at 6.25 μM. EC_50_ = 2.9 μM (0.91 μg/mL) vs. 0.9 μM (0.28 μg/mL) for PZQ. CC_50_ > 200 μM (Vero kidney cells from African green monkey). High selectivity (SI > 68.9).	[227]
	*S. mansoni* (BH strain) In vivo	Female Swiss mice (3-week-old) infected percutaneously with ~80 cercariae an treated with single dose of **20** (400 mg/kg), PZQ (400 mg/kg) or vehicle (2% ethanol in saline), by oral gavage, 21 days (prepatent infection) or 49 days (patent infection) pi. Mice were euthanized 63 days pi.	Reduced worm burden by 52.1% in patent infection. Reduced egg production by 52.3% in patent infection, attenuating hepatomegaly and splenomegaly. Effective against juvenile worms, contrary to PZQ, decreasing worm burden by 52.4% in prepatent infection.	[227]

Abbreviations: BH, Belo Horizonte (Minas Gerais, Brazil); CC_50_ = 50% cytotoxic concentration; CLSM, confocal laser scanning microscopy; DMSO, dimethyl sulfoxide; EC_50_ = 50% effective concentration; i.p., intraperitoneal; LC_50_ = 50% lethal concentration; LE, Luiz Evangelista (Minas Gerais, Brazil); NTS, newly transformed schistosomula; OPG, eggs per gram; pi, post-infection; *p.o.*, *per os* (orally); PZQ, praziquantel; SEM, scanning electron microscopy; SI, selectivity index (CC_50_/EC_50_ ratio).

**Table 4:** Antischistosomal activity of plant-derived diterpenoids.

Compound (Source)	Species (Strain), Type of Study	Treatment	Main Findings	Ref.
Phytol (**21**) as a mixture of isomers (commercially acquired)	*S. mansoni* (BH strain) In vitro	Coupled adult (56-day-old) worm pairs incubated with **21** at 0, 12.5, 25, 50, 75 and 100 μg/mL phytol for 120 h. Positive control: PZQ (1 μg/mL)	Separation of all adult worm pairs after 24 h at 25 μg/mL. Reduced motor activity of female worms at 50 μg/mL, which died after 24 h. Phytol at 100 μg/mL caused 100% mortality of all male and female parasites. CLSM revealed extensive tegumental disruption, with females more susceptible than males. At sublethal conc. (12.5 μg/mL), phytol reduced egg production by 75% in 120 h.	[228]
	*S. haematobium* (Egyptian strain) In vitro	Juvenile (50-day-old) or adult (90-day-old) worms incubated with **21** at 25, 50, 75, 100, 125, and 150 μg/mL or vehicle (0.5% DMSO) for 72 h.	Phytol at 25 μg/mL was inactive. 100% lethality after 48 h at 150 μg/mL (adult worms) or 125 μg/mL (juveniles). Males more susceptible than females. SEM revealed time- and concentration-dependent tegumental damage which correlated with worm viability.	[230]
	*S. mansoni* (BH strain) In vivo	BALB/c mice (3-week-old) infected with 70 cercariae by tail immersion and treated with single oral dose (40 mg/kg) or vehicle (PBS), 56 days pi. Mice were sacrificed 2 weeks post-treatment.	Reduction of total worm burden by 51.2% (70.3% in female worms). Decreased fecal egg counts by 76.6%. Confocal microscopy of adult worms recovered from treated mice revealed tegumental damage, more extensive in females.	[228]
(−)-Sclareol (**22**) (commercially acquired)	*S. mansoni* (PR strain) In vitro	NTS, juvenile (21-day-old) and adult (49-day-old) worms incubated with **22** at 1.65, 3.13, 6.25, 12.5, 25 and 50 μM or vehicle (0.3% DMSO) for 72 h. Positive control: auranofine (10 μM).	Dose-dependent antischistosomal activity with IC_50_ values of 12.3, 5.5, and 19.3 μM against NTS, juvenile, and adult worms, respectively. Reduced egg production at sublethal concentrations. CC_50_ = 74.1 μM (HepG2 human liver cancer cells). SEM and metabolomic analysis of a more potent semi-synthetic derivative supported surface membrane perturbation by interference with arachidonic acid metabolism as a plausible mechanism of action.	[231]
(−)-Polyalthic acid (**23**) (*Copaifera duckei* Dwyer, Fabaceae)	*S. mansoni* (LE strain) In vitro	Adult (49-day-old) worm pairs incubated with **23** at 12.5, 25, 50, 100 and 200 μM or vehicle (0.1% DMSO) for 72 h. Positive control: PZQ (12.5 μM).	Schistosomicidal activity with LC_50_ values of 133.4, 116.3 and 107.9 μM at 24, 48, and 72 h, respectively. Suppression of egg production in 72 h at conc. above 100 μM. Dose-dependent inhibition of egg development. CC_50_ = 409.5 μM (V79 Chinese hamster lung fibroblast cells)	[235]
*ent*-Hardwickiic acid (**24**) (*Copaifera pubiflora*, Fabaceae)	*S. mansoni* In vitro	Adult (49-day-old) worm pairs incubated with **24** at 0, 3.125, 6.25, 12.5, 25, 50, 100 and 200 μM or vehicle (0.1% DMSO) for 72 h. Positive control: PZQ (0.195–1.46 μM)	IC_50_ values of 29.6, 30.8 and 25.7 μM for 24, 48 and 72 h respectively. 100% lethality after 24 h at 200 μM. Extensive tegumental damage was observed in both male and female worms. Separation of 100% of worm pairs at 25 μM and strong inhibition of egg production and egg development at lower sublethal concentrations. Embryogenesis interference was suggested as a possible mechanism of action.	[236]
7-Keto-sempervirol (**25**) (*Lycium chinense*, Solanaceae)	*S. mansoni* (NMRI strain) In vitro	NTS or adult (49-day-old) worm pairs incubated with **25** at 1.56, 3.12, 6.25, 12.5, 25, 50 and 100 μM or vehicle (1% DMSO) for 24 h (NTS) or 72 h (adults). Positive control: auranofin (70 μM).	Dual in vitro activity against *S. mansoni* schistosomula (LD_50_ = 19.1 μM) and *F. hepatica* newly excysted juveniles (LD_50_ = 17.7 μM). Reduced adult *S. mansoni* motility, oviposition, and egg production. SEM revealed extensive tegumental damage in both blood and liver adult flukes treated with **25** at 100 μM for 72 h. CLSM showed irregularly shaped in utero eggs lacking fully formed eggshells and lateral spines in adult *S. mansoni* females treated with **25** at 100 μM for 24 h.	[239]
*ent*-Kaur-16-en-19-oic acid (**26**) (*Baccharis lateralis*, Asteraceae)	*S. mansoni* (BH strain) In vitro	Adult (49-day-old) worm couples incubated with **26** at 0.78, 1.56, 3.12, 6.25, 12.5, 25 and 50 μM or vehicle (0.5% DMSO) for 72 h. Positive control: PZQ (2 μM).	LC_50_ = 26.1 μM at 72 h. 100% lethality after 48 h at 25 μM. Moderate egg load reduction at 25 μM. Treatment of worm couples with **26** did not produce significant alterations in the male and female reproductive organs.	[240]
15β-Senecioyl-oxy-*ent*-kaur-16-en-19-oic acid (**27**) (*Baccharis lateralis*, Asteraceae)	*S. mansoni* (BH strain) In vitro	Adult (49-day-old) worm couples incubated with **27** at 0.78, 1.56, 3.12, 6.25, 12.5, 25 and 50 μM or vehicle (0.5% DMSO) for 72 h. Positive control: PZQ (2 μM).	LC_50_ = 11.6 μM at 72 h. 100% lethality after 24 h at 25 μM. Significant reduction of oviposition after 72 h at 6.25 μM. Decline in egg production associated with loss of male/female pairing. Worm couples treated with **27** showed dilated testes and ovaries. CC_50_ values of 147.2 and 106.2 μM in HaCat human keratinocyte cells and SH-SY5Y human neuroblatoma cells, respectively.	[240]
	*S. mansoni* (BH strain) In vivo	Female Swiss mice (3 weeks old) subcutaneously infected with 80 cercariae and treated with single oral dose (400 mg/kg) or vehicle (0.2% ethanol), 42 days pi. Mice were euthanized 56 pi.	Reduction of total worm burden by 61.9%. Number of immature eggs in intestinal wall and fecal samples decreased by 69.2% and 71.6%, respectively. Achieved a significant reduction in hepatosplenomegaly.	[240]
Pimaradienoic acid (**28**) (*Viguiera arenaria*, Asteraceae)	*S. mansoni* (LE strain) In vitro	Adult (56-day-old) worm couples incubated with **28** at 10, 25, 50 and 100 μM or vehicle (1% DMSO) for 120 h. Positive control: PZQ (10 μM).	Reduced parasite viability in a dose-dependent manner. Produced tegumental alterations at 100 μM within 24 h. Induces 100% separation of coupled adult worms at 100 μM after 24 h, with suppression of egg production. Inhibition of egg development at all conc. tested. CC_50_ > 500 μM (primary human fibroblast cell line).	[241]
	*S. mansoni* (LE strain) In vivo	BALB/c mice infected by percutaneous tail exposure to 60 cercariae for 2 h and treated with single i.p. dose (100 mg/kg) or vehicle (10% DMSO), 49 days pi. Mice were sacrificed 6 days post-treatment.	Reduced total worm burden by 40%.	[241]
12-*O*-acetylphorbol-13-isobutyrate (**29**) (*Croton tiglium*, Euphorbiaceae)	*S. japonicum* In vitro	NTS incubated with **29** at 8.5, 17 and 34 μg/mL or vehicle (DMSO) for 72 h. Positive control: PZQ (30 μg/mL).	100% mortality of NTS after 72 h at 8.5 μg/mL. Ameliorated TGF-β1-induced liver fibrosis in LX-2 cells through regulating the TGF-β1/Smad signaling pathway. IC_50_ = 103.89 μM (human hepatic stellate LX-2 cells).	[19]

Abbreviations: BH, Belo Horizonte (Minas Gerais, Brazil); CC_50_ = 50% cytotoxic concentration; CLSM, confocal laser scanning microscopy; DMSO, dimethyl sulfoxide; IC_50_ = 50% inhibitory concentration; LC_50_ = 50% lethal concentration; LD_50_ = 50% lethal dose; LE, Luiz Evangelista (Minas Gerais, Brazil); NMRI, Naval Medical Research Institute (United States); NTS, newly transformed schistosomula; PBS, phosphate-buffered saline; pi, post-infection; PZQ, praziquantel; SEM, scanning electron microscopy; TGF, transforming growth factor.

**Table 5:** Antischistosomal activity of plant-derived triterpenoids.

Compound (Source)	Species (Strain), Type of Study	Treatment	Main Findings	Ref.
Betulin (**30**) (*Schefflera vinosa* (Cham. & Schltdl.) Frodin, Araliaceae)	*S. mansoni* (LE strain) In vitro	Adult (56-day-old) worm pairs incubated with **30** at 50, 100 and 200 μM or vehicle (1% DMSO) for 120 h. Positive control: PZQ (12.5 μM).	Reduced motor activity by 50% at 100 μM. 25% lethality after 24 h at 200 μM, achieving 50% after 120 h. No tegumental damage observed.	[243]
Oleanolic acid (**31**) (*Miconia langsdorffii*, Melastomataceae)	*S. mansoni* (LE strain) In vitro	Adult (56-day-old) worm pairs incubated with **31** at 50, 100 and 200 μM or vehicle (1% DMSO) for 120 h. Positive control: PZQ (12.5 μM).	Reduced motor activity by 25% at 200 μM. No tegumental damage observed.	[243]
Ursolic acid (**32**) (*Miconia langsdorffii*, Melastomataceae)	*S. mansoni* (LE strain) In vitro	Adult (56-day-old) worm pairs incubated with **32** at 50, 100 and 200 μM or vehicle (1% DMSO) for 120 h. Positive control: PZQ (12.5 μM).	Reduced motor activity by 25% at 50 μM. No tegumental damage observed.	[243]
Betulinic acid (**33**) (*Eremanthus erythropappus* DC. McLeisch, Asteraceae)	*S. mansoni* (BH strain) In vitro	Paired male and female adult worms (49-day-old) exposed to **33** at 25, 50, 100 and 200 μg/mL or vehicle (0.5% DMSO) for 72 h. Positive control: PZQ (0.62 μg/mL).	EC_50_ = 36.8 μM at 72 h. CC_50_ > 500 μM (Vero kidney cells from African green monkey). Good selectivity (SI > 15.5). Light microscopy showed no tegumental alterations.	[172]
	*S. mansoni* (BH strain) In vivo	Female Swiss mice (3-week-old) percutaneously infected with 80 cercariae and treated with single oral dose of **33** (400 mg/kg), PZQ (400 mg/kg) or vehicle (2% ethanol in saline), 49 days pi. Mice were euthanized 63 days pi.	Reduction of total worm burden by 41.9%. Less effective than PZQ, which showed 92.6% worm burden reduction.	[172]
Balsaminol F (**34**) (*Momordica balsamina* L., Cucurbitaceae)	*S. mansoni* (LE strain) In vitro	Adult (56-day-old) worm pairs exposed to **34** at 10, 25, 50 and 100 μM or vehicle (1% DMSO). Positive control: PZQ (10 μM).	Moderate schistosomicidal activity (LC_50_ = 14.7 ± 1.5 μM at 24 h). 100% lethality after 24 h at 50 μM. Separation of all worm pairs after 24 h at 25 μM. Significant reduction in worm motor activity and egg production at ≥10 μM. Additional antimalarial activity against blood schizonts of *Plasmodium falciparum* chloroquine-sensitive (IC_50_ = 18.0 ± 1.7 μM) and -resistant (IC_50_ = 20.0 ± 4.3 μM) strains. CC_50_ = 27.3 ± 1.2 μM (human breast cancer MCF7 cells).	[250,252]
Karavilagenin C (**35**) (*Momordica balsamina* L., Cucurbitaceae)	*S. mansoni* (LE strain) In vitro	Adult (56-day-old) worm pairs exposed to **35** at 10, 25, 50 and 100 μM or vehicle (1% DMSO). Positive control: PZQ (10 μM).	Moderate schistosomicidal activity (LC_50_ = 28.9 ± 1.8 μM at 24 h). 100% lethality after 24 h at 100 μM. Separation of all worm pairs after 24 h at 50 μM. Significant reduction in worm motor activity and egg production at ≥25 μM. Additional antimalarial activity against blood schizonts of *Plasmodium falciparum* chloroquine-sensitive (IC_50_ = 10.4 ± 0.7 μM) and -resistant (IC_50_ = 11.2 ± 0.7 μM) strains. CC_50_ = 16.7 ± 2.1 μM (human breast cancer MCF7 cells).	[251,252]
Limonin (**36**) (dried seeds of bittersweet orange, *Citrus x aurantium*, Rutaceae)	*S. mansoni* (Egyptian strain) In vivo	Male Swiss albino mice CD-1 (3-week-old) subcutaneously infected with ~100 cercariae and treated with single dose of **36** (50 or 100 mg/kg) or vehicle (10 mg/mL gum acacia solution in saline) by orogastric gavage, 21-days or 56-days pi. Positive control: PZQ (500 mg/kg/day in 2% Cremophor-EL), for 2 days. Mice were sacrificed 2 weeks post-treatment.	Reduction of total worm burden in juveniles (by 70% at 50 mg/kg and 83.3% at 100 mg/kg) and adult worms (by 41.1% at 50 mg/kg and 60.3% at 100 mg/kg). Juvenile female worms more susceptible than males but the opposite pattern was observed in adult stages. SEM revealed extensive tegumental damage to both juvenile and adult male worms. Egg load reduction in liver (34.9% at 50 mg/kg and 47.2% at 100 mg/kg) and intestine (46.7% at 50 mg/kg and 56.1% at 100 mg/kg) in the adult-treated groups. Reduction in both size and number of granulomas, ameliorating infection-induced hepatic fibrosis.	[260]
23-Hydroxy-3-oxo-9β-lanosta-7,24-dien-26,23-olide (**37**) (*Abies procera*, Pinaceae)	*S. mansoni* (PR strain) In vitro	NTS, juvenile (21-day-old) and adult (49-day-old) worms exposed to **37** at 0.62, 1.25, 2.5, 5, 10 and 20 μM or vehicle (1.25% DMSO) for 72 h. Positive controls: PZQ (10 μM), auranofin (10 μM).	Higher efficacy against NTS (EC_50_ = 1.9 μM) and juveniles (EC_50_ = 3.4 μM). Adult males more susceptible than females (EC_50_ = 7.4 μM vs. 10.3 μM). Egg production inhibition at sublethal concentrations correlated with loss of male–female pairing. SEM showed tegumental disruption and membrane blebbing. CC_50_ values of 32 and 33 μM in human liver cancer HepG2 cells and madin darby bovine kidney MDBK cells, respectively.	[264]

Abbreviations: BH, Belo Horizonte (Minas Gerais, Brazil); CC_50_ = 50% cytotoxic concentration; DMSO, dimethyl sulfoxide; EC_50_ = 50% effective concentration; IC_50_ = 50% inhibitory concentration; LC_50_ = 50% lethal concentration; LE, Luiz Evangelista (Minas Gerais, Brazil); NTS, newly transformed schistosomula; pi, post-infection; PZQ, praziquantel; SEM, scanning electron microscopy.

**Table 6:** Antischistosomal activity of plant-derived triterpenoid saponins.

Compound (Source)	Species (Strain), Type of Study	Treatment	Main Findings	Ref.
Hederacolchiside A1 (**38**) (*Pulsatilla chinensis* (Bunge) Regel, Ranunculaceae)	*S. japonicum* In vivo	ICR mice percutaneously infected with ~65 cercariae and treated with **38** (8 mg/kg/day), i.p., for 5 consecutive days starting at day 1, 7, 14, 21, 28 or 35 pi. Mice were euthanized 49 days pi.	Significant and comparable total and female worm burden reductions in mice infected with either juvenile or adult worms. More potent than positive controls in NTS-infected mice, reducing total, female, and egg loads by 97.2%, 92.4%, and 99.3%, respectively. Ameliorated liver damage, decreasing serum levels of inflammatory cytokines (TNF-α, IL-4, IL-17A) and expression of fibrotic proteins (Col I, TGF-β1, TIMP-1) in the mouse liver.	[271]
**38**	*S. mansoni* In vivo	ICR mice subcutaneously infected with ~80 cercariae and treated with **38** (8 mg/kg/day), i.p., for 5 consecutive days starting at day 1, 7, 21, 42 and 49 pi. Mice were euthanized 56 days pi.	Significant and comparable total and female worm burden reductions in mice infected with either juvenile or adult worms. More active against 1-day-old and 7-day-old schistosomes, achieving total worm burden reduction of 88.6% and 80.7%, respectively, compared with 68% for 21-day-old juveniles and 84.1% for 49-day-old adult worms.	[271]
Hederacolchiside C (**39**) (*Pulsatilla chinensis* (Bunge) Regel, Ranunculaceae)	*S. japonicum* (Anhui strain) In vitro	Adult (42-day-old) worm pairs incubated with **39** at 3.75, 7.5, 15, 30 and 60 μg/mL for 72 h. Positive controls: PZQ (30 μg/mL), AS (30 μg/mL).	100% lethality after 72 h at 60 μg/mL. SEM revealed swelling and peeling of the tegument, more pronounced in female worms.	[273]
**39**	*S. japonicum* (Anhui strain) In vivo	Female ICR mice infected by direct skin contact through exposure to 55 ± 5 cercariae and treated with **39** (100, 200 or 400 mg/kg, bid, i.v.), PZQ (300 mg/kg, *p.o.*) or AS (300 mg/kg, *p.o.*) for 5 days, at 14–18 or 35–39 days pi. Mice were sacrificed 49 days pi.	Dose-dependent reductions in total worm burden (28.9–44.1% for juveniles and 27.9–48.1% for adult worms). Females more susceptible than males, with reduction rates of 30.7–49.2% in juveniles and 38.7–62.5% in adults. Hepatic egg load reductions by 39.9–58.6% in juveniles and 25.5–63.5% in adults. **39** treatment reduced granuloma size and decreased serum levels of IgG, TNF-α, IL-4 and IL-17A involved in the granulomatous inflammatory response.	[273]
Asiaticoside (**40**) (*Centella erecta*, Apiaceae)	*S. mansoni* (BH strain) In vivo	Female Swiss mice (3-week-old) subcutaneously infected with 80 cercariae and treated with single dose of **40** (400 mg/kg), PZQ (400 mg/kg) or vehicle (2% *v*/*v* ethanol in PBS), by oral gavage, 49 days pi. Mice were euthanized 63 days pi.	Reduction of total worm burden by 65.4% vs. 93.1% for PZQ. Reduction of fecal egg load by 67.7% vs. 93.6% for PZQ. Oogram pattern showed reduction in immature eggs by 71.5% vs. 94.4% for PZQ.	[275]

Abbreviations: AS, artesunate; BH, Belo Horizonte (Minas Gerais, Brazil); bid, twice daily; Col, collagen; ICR, Institute of Cancer Research; IgG, immunoglobulin G; IL, interleukin; i.v., intravenous; NTS, newly transformed schistosomula; PBS, phosphate-buffered saline; pi, post-infection; *p.o.*, *per os* (orally); PZQ, praziquantel; SEM, scanning electron microscopy; TGF, transforming growth factor; TIMP, tissue inhibitor of metalloproteinases; TNF, tumor necrosis factor.

## Data Availability

No new data were created or analyzed in this study.

## Abbreviations

The following abbreviations are used in this manuscript:

ART	Artemisinin
ACTs	ART-based combination therapies
AM	Artemether
AS	Artesunate
CVNE	Carvacrol Nanoemulsion
CANE	Carvacryl acetate nanoemulsion
DHA	Dihydroartemisinin
EOs	Essential oils
HSCs	Hepatic stellate cells
i.p.	intraperitoneal
i.v.	intravenous
MDA	Mass drug administration
NLCs	Nanostructured lipid carriers
NTDs	Neglected Tropical Diseases
OPG	Eggs per gram
PZQ	Praziquantel
ROS	Reactive oxygen species
SEAs	Soluble egg antigens
STLs	Sesquiterpene lactones
SLNs	Solid lipid nanoparticles
TRP	Transient receptor potential
